# Supplementary material for: The transcription factor ZEB1 regulates stem cell self-renewal and cell fate in the adult hippocampus
Source: Cell Rep. 2021 Aug 24;36(8):109588. doi: 10.1016/j.celrep.2021.109588 (PMC8411115; doi:10.1016/j.celrep.2021.109588)
Supplement: Document S1. Figures S1–S5 and Tables S1–S3 [file mmc1.pdf]

Cell Reports, Volume *36*

Supplemental information

The transcription factor ZEB1 regulates stem cell self-renewal and cell fate in the adult hippocampus

Bhavana Gupta, Adam C. Errington, Ana Jimenez-Pascual, Vasileios Eftychidis, Simone Brabletz, Marc P. Stemmler, Thomas Brabletz, David Petrik, and Florian A. Siebzehnrubl

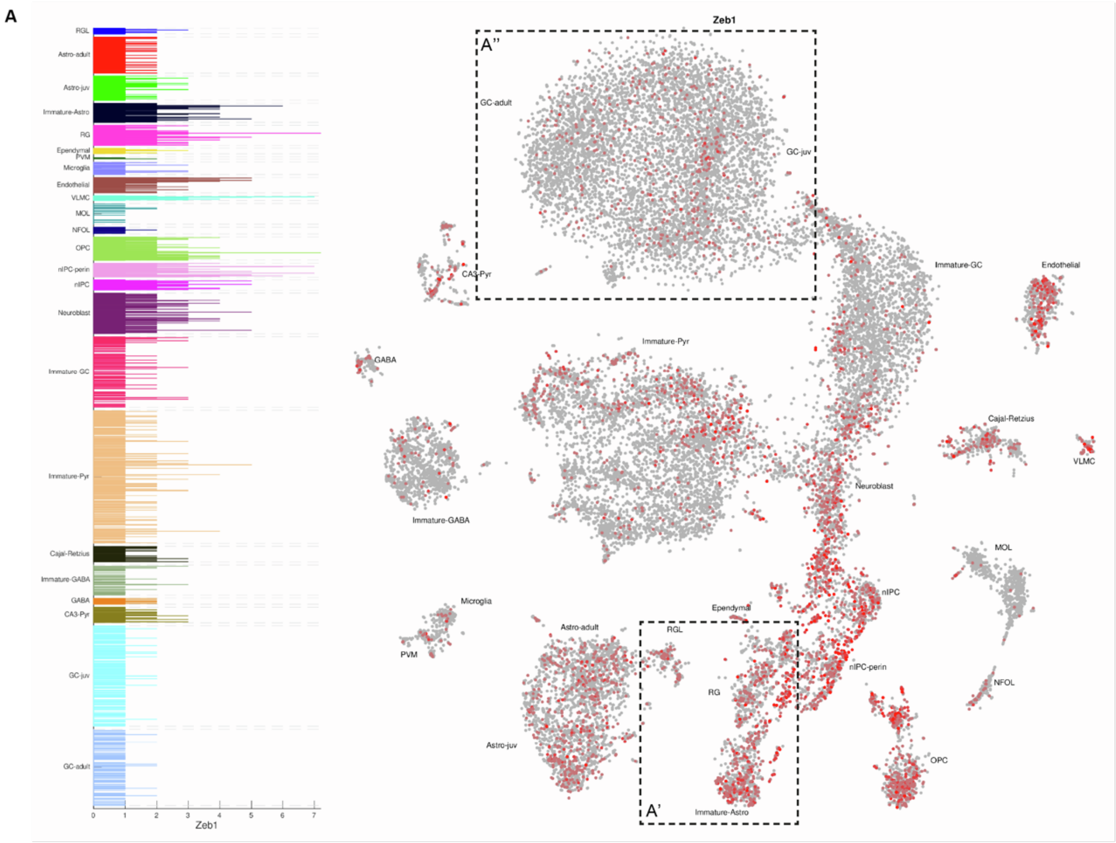

Figure S1: Single-cell RNA-Sequencing data of *Zeb1* expression across hippocampal cell types. Related to Figure 1. Depicted is data from Hochgerner et al. (Hochgerner et al., 2018) based on 10X Genomics sequencing. Boxed areas indicate RGL cell and immature astrocyte populations **(A')** as well as hippocampal granule cells **(A'')**.

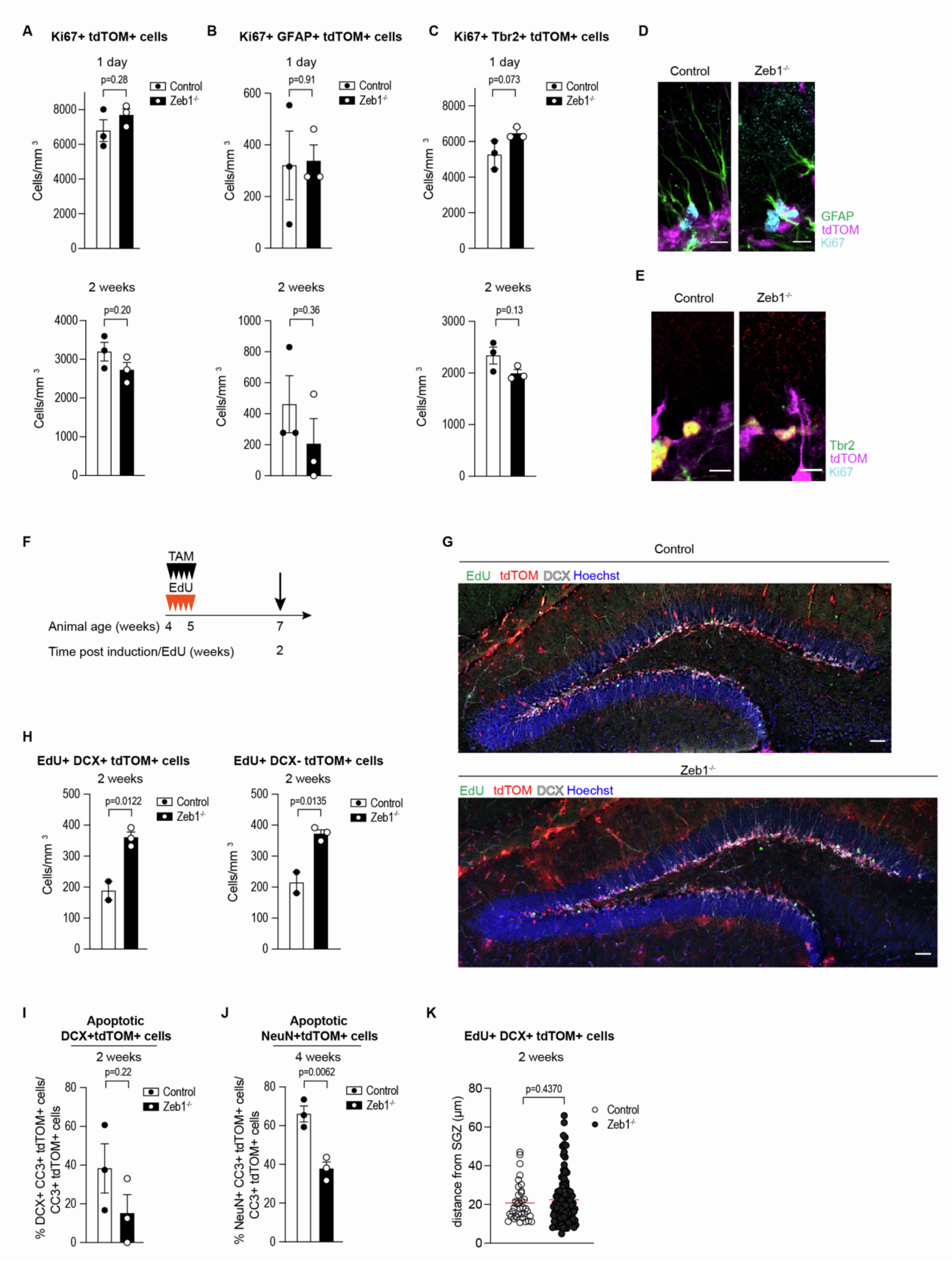

Figure S2: *Zeb1* deletion does not result in slower proliferation rates but specifically increases neuronal survival. Related to Figure 4. (A) Quantification of proliferating Ki67+ cells in control and *Zeb1*$^{-/-}$ mice. Both genotypes show no significant differences at 1 day or 2 weeks post induction. **(B)** Quantification of proliferating Ki67+GFAP+tdTOM+ RGL cells in control and *Zeb1*$^{-/-}$ mice. Both genotypes show no significant differences at 1 day or 2 weeks post induction. **(C)** Quantification of proliferating Ki67+Tbr2+tdTOM+ IPCs in control and *Zeb1*$^{-/-}$ mice. Both genotypes

show no significant differences at 1 day or 2 weeks post induction. **(D-E)** Representative images of Ki67+GFAP+tdTOM+ cells **(D)** and Ki67+Tbr2+tdTOM+ at 2 weeks post-induction **(E)**. Scale bars 10 µm. **(F)** Schematic of EdU administration concurrent with tamoxifen; tissue harvesting was carried out at 2 weeks post-EdU injections. **(G)** Representative images of hippocampi stained for EdU, DCX and tdTOM. Scale bars 100 µm. **(H)** Quantification of EdU+ cells showed a significant increase of both EdU+tdTOM+DCX+ (left) and EdU+tdTOM+DCX- (right) cells 2 weeks after EdU injection. **(I)** Quantification of the fraction of apoptotic CC3+ neuroblasts (DCX+) showed no difference between the genotypes. **(J)** Quantification of the fraction of apoptotic CC3+ neurons (NeuN+) demonstrated a decrease in the number of apoptotic neurons in the $Zeb1^{-/-}$ mice in comparison to controls at 4 weeks post-induction. **(K)** Distances of EdU+tdTOM+DCX+ neuroblasts from the SGZ show no significant differences in migration of newborn neuroblasts between genotypes. Dots represent individual animals, except in K where dots represent individual cells.

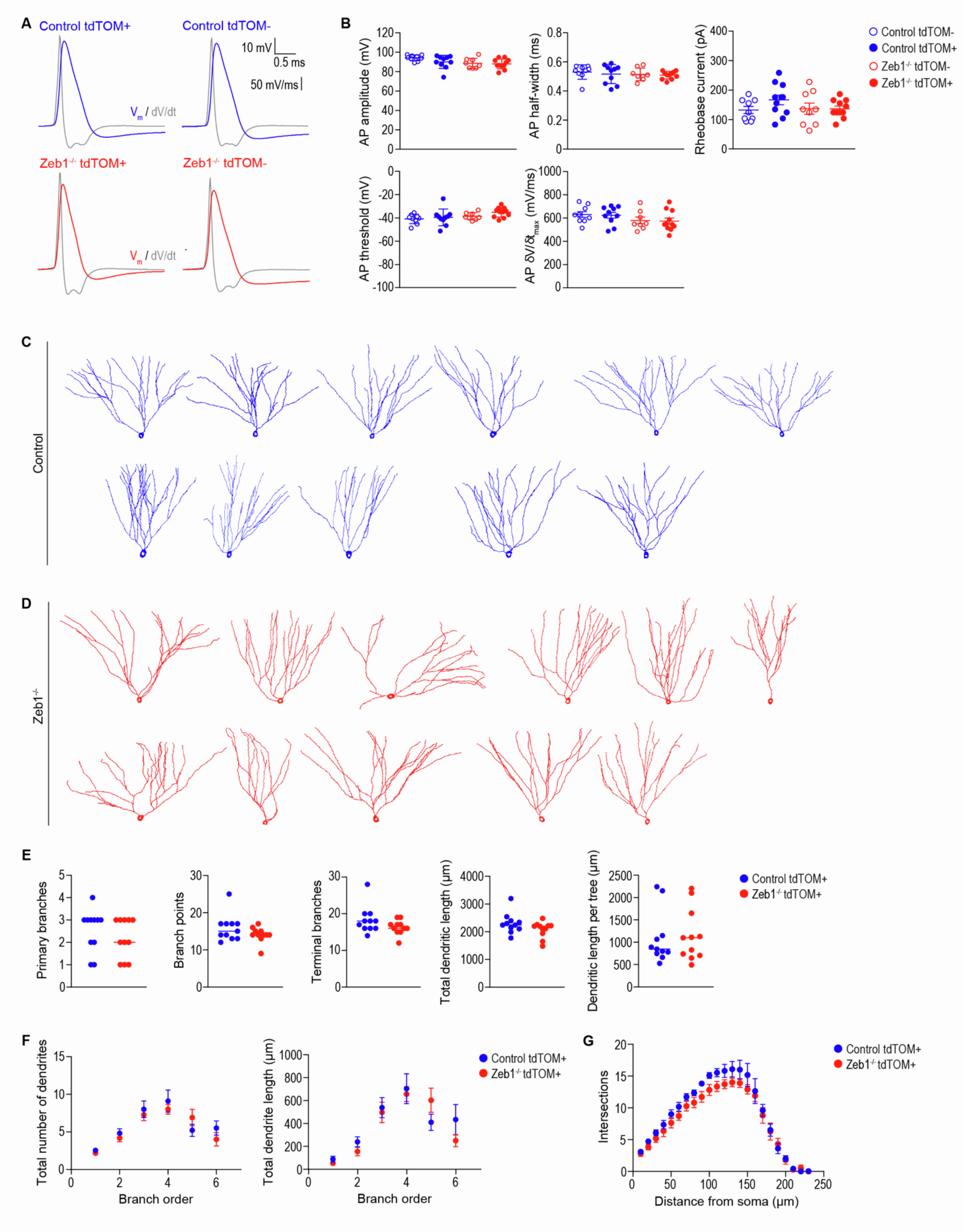

Figure S3: Patch-clamp electrophysiology of control and *Zeb1*$^{-/-}$ neurons. Related to Figure 4.

(A) Traces showing evoked action potentials, in tdTOM+ and tdTOM-, control (blue) and *Zeb1*$^{-/-}$ (red) DGGCs. Grey traces show the first temporal derivative (δV/δt) of the action potential. **(B)** Scatter

plots showing action potential amplitude, half width, voltage threshold, δV/δt and rheobase current for individual DGGCs overlaid with the mean and SEM value for each group. (**C-D**) 2D projections of reconstructed DGGCs from control **(C)** and Zeb1$^{-/-}$ mice (**D**, n = 11 each). **(E-F)** Plots of the mean number of dendrites and mean total dendritic length by branch order in control and *Zeb1*$^{-/-}$ DGGCs. **(G)** Sholl plot of the number of dendritic intersections against distance from the soma. Dots represent individual neurons in B and E, and averages in F and G.

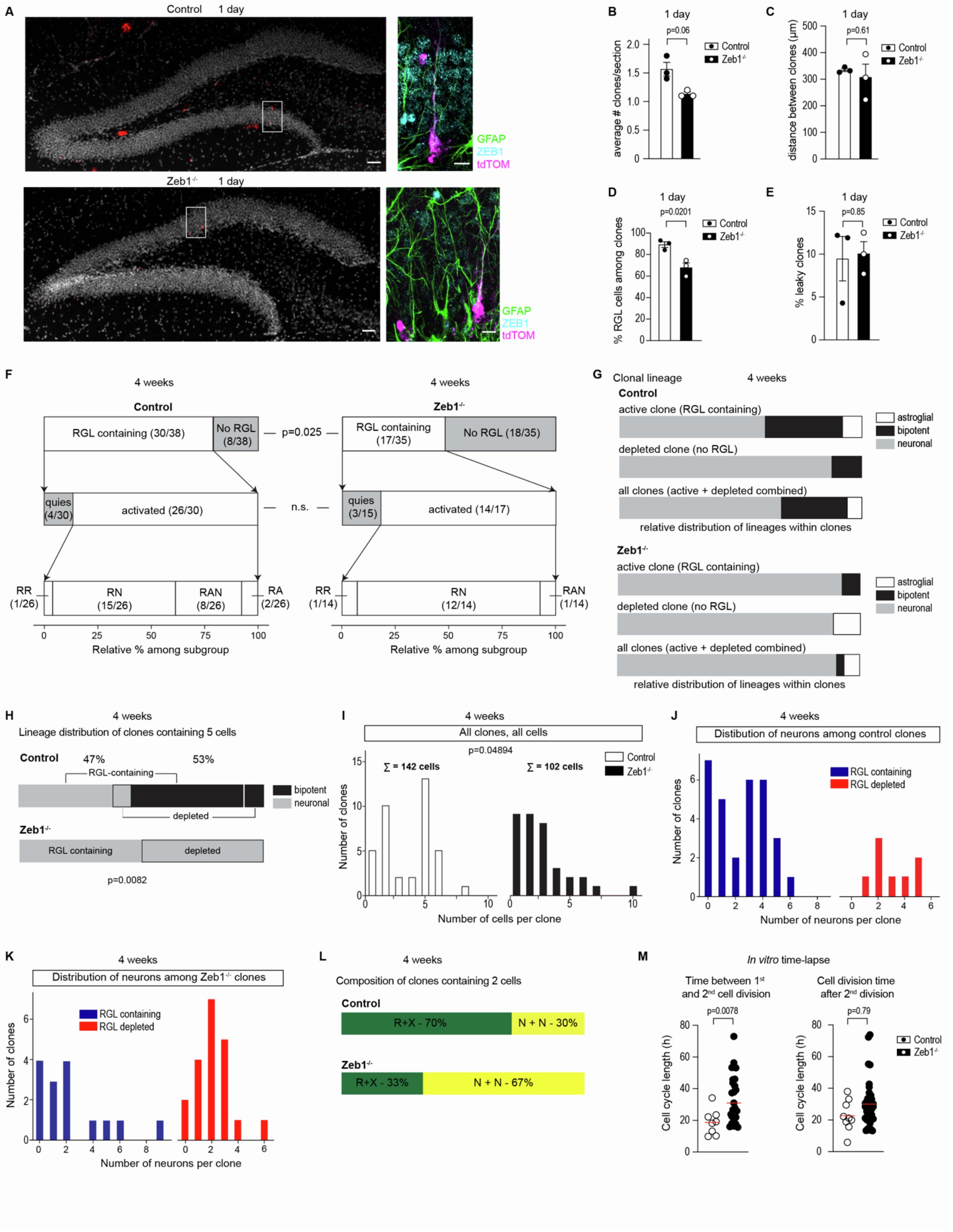

Figure S4: Additional data from clonal analysis. Related to Figure 6. (A) Representative images of hippocampi 1 day after administration of low-dose tamoxifen. Insets show recombination of tdTOM in RGL cells (ZEB1+ in control, ZEB1- in *Zeb1*$^{-/-}$). Note the presence of the tdTOM+ IPC in proximity

to the RGL cell in *Zeb1*$^{-/-}$. Recombined clones in *Zeb1*$^{-/-}$ mice displayed evidence of accelerated proliferation compared to controls (see also D). Scale bars 100 μm (10 μm in insets). **(B)** Quantification of recombined clones per section 1 day after induction shows no difference between genotypes. **(C)** Average distance between recombined clones (where there was more than 1 clone per section) shows no difference between genotypes. **(D)** Quantification of numbers of RGL cells per clone shows a significant decrease in *Zeb1*$^{-/-}$ mice compared to controls at 1 day post induction. **(E)** Quantification of leaky clones (containing >4 cells at 1 day post induction) shows no difference between genotypes. **(F)** Comparison of clonal distribution shows significantly fewer active (RGL cell-containing) clones in *Zeb1*$^{-/-}$ mice. Among RGL-containing (active) clones, there is no difference in the ratio of quiescent (containing only a single RGL cell) to activated (containing an RGL cell and any other cell) clones. Lineage distribution of progenies in activated clones showed a profound loss of astroglial cells in *Zeb1*$^{-/-}$ clones (R: RGL cell; RR: self-renewing clone containing 2 RGL cells; RN: clone containing RGL cell and neuronal progenies; RA: clone containing RGL cell and astroglial progenies; RAN: clone containing RGL cell, neuronal and astroglial progenies). **(G)** Comparison of lineage distribution across active, depleted and all clones highlights increase in neuron-producing and decease in astrocyte-producing clones in *Zeb1*$^{-/-}$ mice. No difference was found between the percentages of neuronal lineage producing depleted clones, but these were far fewer in total numbers in control mice. **(H)** Comparison of lineage distribution across clones containing 5 cells. In controls, 53% of 5-cell clones are bipotent (producing neuronal and astroglial progenies), whereas in Zeb1$^{-/-}$ mice all 5-cell clones were committed to the neuronal lineage. In controls, the majority of 5-cell clones contained an RGL cell, but in *Zeb1*$^{-/-}$ mice the number of active and depleted 5-cell clones was approximately even. **(I)** Total number of cells per clone was significantly lower in *Zeb1*$^{-/-}$ clones compared to controls. Note the different distribution of cells among clones between controls (peaks at clones containing either 2 or 5 cells) and *Zeb1*$^{-/-}$ mice (left shift towards clones containing 1-3 cells). **(J,K)** Histograms showing distribution of neurons between RGL-containing and RGL-depleted clones for control **(J)** and Zeb1$^{-/-}$ mice **(K)**. In controls, most clones contain an RGL cell with peaks at 1 and 4 neurons (corresponding to peaks at 2 and 5 cells in panel D), whereas most *Zeb1*$^{-/-}$ clones lack an RGL cell with a single peak around 2 neurons. **(L)** Comparison of lineage distribution across clones containing 2 cells. In control mice, most 2-cell clones are activated,

whereas in *Zeb1*$^{-/-}$ mice the majority of 2-cell clones is depleted and contains neurons. **(M)** Quantification of cell division times between first and second division (left) and all subsequent divisions (right) from time-lapse imaging. Red line represents median. Dots represent individual animals in B-E and individual clones in M.

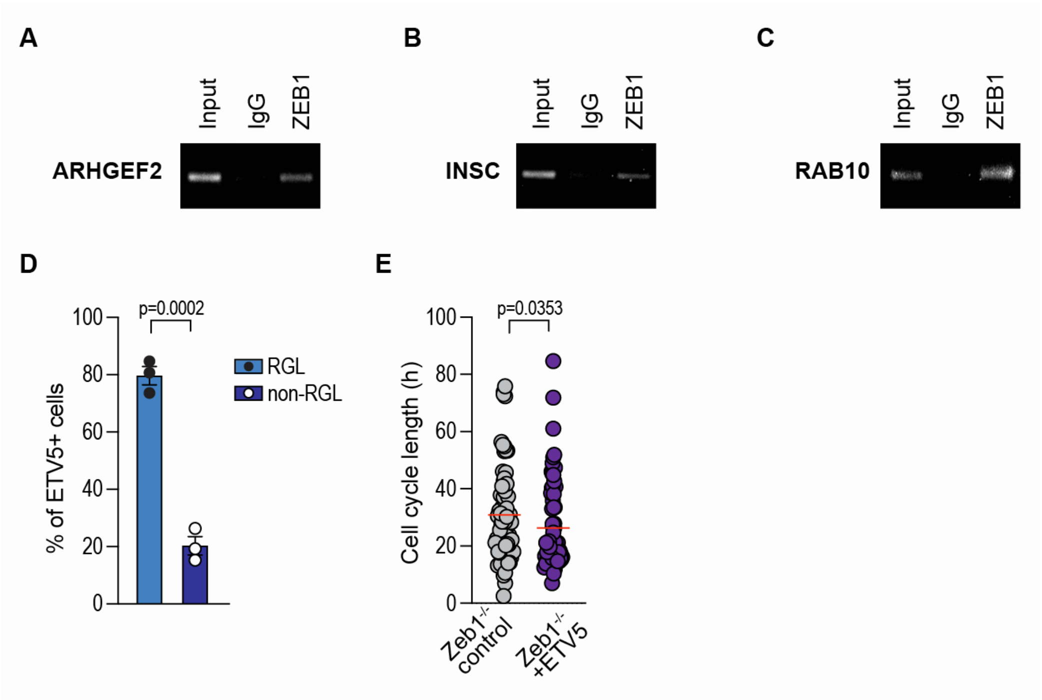

Figure S5: ChIP data for promoter occupancy of ZEB1. Related to Figure 7. (A-C) ChIP-PCR demonstrated ZEB1 binding to the promoter regions of ARHGEF2 **(A)**, INSC **(B)** and RAB10 **(C)**. **(D)** Quantification of ETV5 expression across cell types in the SGZ shows that the majority of ETV5 expressing cells are RGL cells, with a small fraction of non-RGL ETV5+ cells. **(E)** Quantification of cell cycle length in *Zeb1*$^{-/-}$ control and ETV5-transduced clones. Red line represents median. Dots represent individual animals in D and individual cells in E.

Table S1: Candidate regulators of asymmetric cell division. Candidates included in further analyses are highlighted. Related to Figure 7.

**Candidate gene name**	ACTR2	ACTR3	ARHGEF2	ASPM	DOCK7	ETV5	FGF13	GOLGA2	HOXC4	ING2	INSC	PARD3	PAX6	POU5F1	RAB10	RGS14	SOX5	STRA8	TEAD3	WNT9B	ZBTB16
ZEB1 bound in GBM ChIP-Seq?		♦	♦			♦			♦	♦	♦	♦			♦		♦				
Expression in HC cell types (1)	neuron	neuron	IPC/ neuron	IPC	astro, neuron	RGL, astro, OPC	neuron	broad		broad	neuroblast	broad	RGL, astro, neuroblast		broad	granule cell	broad				Neuron
Expression in astrocytes (2)				♦ (STR)		♦ (HC)					♦ (STR)	♦ (STR)	♦ (HC)								♦ (HC)
Cell types showing overlap with ZEB1 (3)	oligo	oligo	astro/OPC		oligo	astro/OPC	oligo	astro/ OPC		oligo	astro	astro	astro		astro/ oligo	oligo	astro/ OPC				astro/ OPC

(1) Hochgerner et al. Nat Neurosci 2018 DOI: 10.1038/s41593-017-00562-2
(2) Chai et al. Neuron 2017 DOI: 10.1016/j.neuron.2017.06.029
(3) Zeisel et al. Science 2015 DOI: 10.1126/science.aaa1934

Acronyms:
astro = astrocyte
GBM = glioblastoma
HC = hippocampus
IPC = intermediate progenitor cell
oligo = oligodendrocyte
OPC = oligodendrocyte precursor cell
RGL = radial glia like cell
STR = striatum

Table S2: Criteria for identifying cell types in the DG. Related to STAR Methods sections ‘Tissue processing, immunostaining, and confocal imaging’ and ‘Clonal analysis’.

Cell population	Markers	Localization/Morphological features
RGL cell	GFAP	SGZ-localized soma, a radial projection spanning the GCL and branching in ML
Activated RGL, early IPC	MCM2	SGZ-localized, either GFAP+ RGL cell-like morphology or spherical morphology and GFAP- (IPC)
Proliferating IPC	Ki67	SGZ-localized, spherical
Late IPC	TBR2	SGZ-localized, spherical with some short processes
Neuroblast, immature neuron	DCX	SGZ and lower GL-localized, short process extending towards upper GCL
Mature neuron	NeuN	Pan-DG localization with a projection branching in the ML
Astrocyte	GFAP, S100ß	SGZ, hilus, and ML-localized with a stellate morphology

Table S3: Oligonucleotide information, related to STAR Methods.

Reagent or Resource	Source	Identifier
ARHGEF fwd #1 (ChIP)	Sigma-Aldrich	TCTGGCTTGTGTGGCTGAAA
ARHGEF rev #1 (ChIP)	Sigma-Aldrich	TCACAAATCAGAGCCCGGTT
ARHGEF fwd #2 (ChIP)	Sigma-Aldrich	GTCTGTGTGGGCAAAACACG
ARHGEF rev #2 (ChIP)	Sigma-Aldrich	AGCTAATCCCAACTGGAGCC
ETV5 fwd #1 (ChIP)	Sigma-Aldrich	ATTCAGGCAAGAGGGGGAGT
ETV5 rev #1 (ChIP)	Sigma-Aldrich	ACAACATACTGGGCGAGCCA
ETV5 fwd #2 (ChIP)	Sigma-Aldrich	TGCACAATGGGGTCTGGTTA
ETV5 rev #2 (ChIP)	Sigma-Aldrich	CCCCTCTTGCCTGAATGGTG
INSC fwd #1 (ChIP)	Sigma-Aldrich	TCCAGAGAGTACAGGCCACA
INSC rev #1 (ChIP)	Sigma-Aldrich	TCACCAATACCACGCCAGAG
INSC fwd #2 (ChIP)	Sigma-Aldrich	GAAGCACATGCTGATCTGTGG
INSC rev #2 (ChIP)	Sigma-Aldrich	AGGAAGCCGCCTCACCAATA
PARD3 fwd #1 (ChIP)	Sigma-Aldrich	GAGCTCCACCTGCCTTTGAC
PARD3 rev #1 (ChIP)	Sigma-Aldrich	AAAGAAGTTGTGCCTGGCTTG
RAB10 fwd #1 (ChIP)	Sigma-Aldrich	GGCCTCCTGATTGGGTGAG
RAB10 rev #1 (ChIP)	Sigma-Aldrich	CTATACGGACGTAGTGCGCC
RAB10 fwd #2 (ChIP)	Sigma-Aldrich	AGAGCCACCTTTTTCCCCGT
RAB10 rev #2 (ChIP)	Sigma-Aldrich	GGTGTACGTCCTTGCGTGC
SOX5 fwd #1 (ChIP)	Sigma-Aldrich	AGCGAAGTGGAGCTATGACA
SOX5 rev #1 (ChIP)	Sigma-Aldrich	GAGCGCTCTCAGCAGTAAGT
SOX5 fwd #2 (ChIP)	Sigma-Aldrich	TGGAGCTATGACATCCCACAT
SOX5 rev #2 (ChIP)	Sigma-Aldrich	AGCGCTCTCAGCAGTAAGTA
