# Supplementary material for: The transcription factor ZEB1 regulates stem cell self-renewal and cell fate in the adult hippocampus
Source: Cell Rep. 2021 Aug 24;36(8):109588. doi: 10.1016/j.celrep.2021.109588 (PMC8411115; doi:10.1016/j.celrep.2021.109588)
Supplement: Document S2. Article plus supplemental information [file mmc2.pdf]

Cell Reports

Article

The transcription factor ZEB1 regulates stem cell self-renewal and cell fate in the adult hippocampus

Graphical abstract

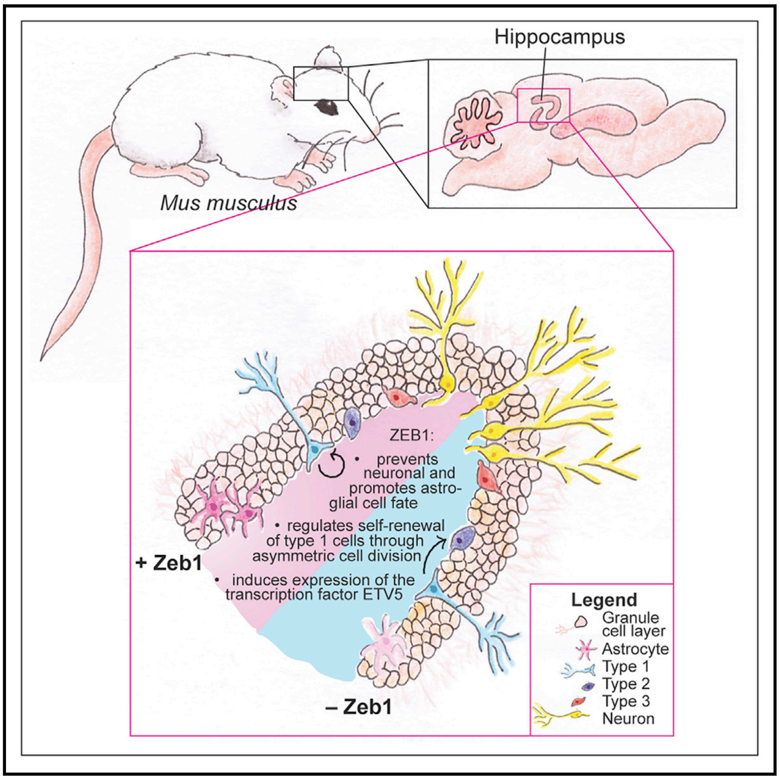

Authors

Bhavana Gupta, Adam C. Errington, Ana Jimenez-Pascual, ..., Thomas Brabletz, David Petrik, Florian A. Siebzehnrubl

Correspondence

fas@cardiff.ac.uk

In brief

Gupta et al. show that the stem cell transcription factor, ZEB1, labels activated hippocampal stem cells and regulates their self-renewal and astroglial fate specification. Mechanistically, ZEB1 promotes asymmetric cell divisions and induces the expression of ETV5, a transcriptional regulator of asymmetric divisions and astroglial lineage commitment.

Highlights

- The stem cell transcription factor, ZEB1, demarcates active from quiescent RGL cells
- ZEB1 drives the self-renewal of RGL cells by promoting asymmetric cell division
- Loss of *Zeb1* causes increased neurogenesis and decreased astrogliogenesis
- ZEB1 induces *Etv5* expression to regulate asymmetric cell division

Gupta et al., 2021, Cell Reports *36*, 109588
August 24, 2021 © 2021 The Author(s).
https://doi.org/10.1016/j.celrep.2021.109588

CellPress

Cell Reports

CellPress
OPEN ACCESS

Article

The transcription factor ZEB1 regulates stem cell self-renewal and cell fate in the adult hippocampus

Bhavana Gupta,[1] Adam C. Errington,[2] Ana Jimenez-Pascual,[1] Vasileios Eftychidis,[1] Simone Brabletz,[3] Marc P. Stemmler,[3] Thomas Brabletz,[3] David Petrik,[4] and Florian A. Siebzehnrubl[1,5,*]

[1]European Cancer Stem Cell Research Institute, Cardiff University School of Biosciences, Cardiff CF24 4HQ, UK
[2]Neuroscience and Mental Health Research Institute, Cardiff University School of Biosciences, Cardiff CF24 4HQ, UK
[3]Department of Experimental Medicine I, Friedrich Alexander University Erlangen-Nuremberg, 91054 Erlangen, Germany
[4]Cardiff University School of Biosciences, Cardiff CF10 3AX, UK
[5]Lead contact
*Correspondence: fas@cardiff.ac.uk
https://doi.org/10.1016/j.celrep.2021.109588

SUMMARY

Radial glia-like (RGL) stem cells persist in the adult mammalian hippocampus, where they generate new neurons and astrocytes throughout life. The process of adult neurogenesis is well documented, but cell-autonomous factors regulating neuronal and astroglial differentiation are incompletely understood. Here, we evaluate the functions of the transcription factor zinc-finger E-box binding homeobox 1 (ZEB1) in adult hippocampal RGL cells using a conditional-inducible mouse model. We find that ZEB1 is necessary for self-renewal of active RGL cells. Genetic deletion of *Zeb1* causes a shift toward symmetric cell division that consumes the RGL cell and generates pro-neuronal progenies, resulting in an increase of newborn neurons and a decrease of newly generated astrocytes. We identify ZEB1 as positive regulator of the ets-domain transcription factor ETV5 that is critical for asymmetric division.

INTRODUCTION

Neural stem cells persist in the adult hippocampus across many mammalian species (Gage, 2019; Ming and Song, 2011), including humans (Coras et al., 2010; Kempermann et al., 2018). Radial glia-like (RGL) cells within the subgranular zone (SGZ) of the dentate gyrus (DG) reside in a quiescent state and undergo self-renewal, or differentiate into neurons or astrocytes upon activation. During neurogenesis, the process of neuron production, RGL cells give rise to intermediate progenitor cells (IPCs) (Kempermann et al., 2004). IPCs can clonally expand (Pilz et al., 2018) and commit to the neuronal lineage to become neuroblasts, which mature into granule neurons that incorporate into the DG circuitry. RGL cell numbers decrease with age (Encinas et al., 2011; Martín-Suárez et al., 2019), but what remains unclear is whether this is due to a limited number of cell divisions per RGL cell (Encinas et al., 2011), or whether the RGL cell pool is sustained over the lifetime of an animal, with a slight age-related decline (Bonaguidi et al., 2011). Likewise, it is not yet fully established whether astrogliogenesis in the DG occurs through terminal differentiation of RGL cells (Encinas et al., 2011), concurrently with neurogenesis (Bonaguidi et al., 2011), through as-yet-unidentified astrocyte-specific RGL cells, or through a combination of all three.

Transcription factors that regulate astroglial versus neuronal specification have been identified (Bonzano et al., 2018; White et al., 2020), but transcriptional mechanisms underpinning the choice between self-renewal and lineage commitment in the adult brain remain incompletely understood.

Zinc-finger E-box binding homeobox 1 (ZEB1) is one of two members of the ZEB transcription factor family, which regulate stem cell self-renewal and epithelial-mesenchymal transition (EMT) in solid tissues (Goossens et al., 2017; Stemmler et al., 2019). Through these functions, ZEB1 also promotes malignant growth and dissemination of brain tumors (Edwards et al., 2011; Rosmaninho et al., 2018; Siebzehnrubl et al., 2013). ZEB1 acts either as transcriptional activator or repressor depending on the recruited cofactors (Rosmaninho et al., 2018; Spaderna et al., 2008). ZEB1 expression is crucial for the maintenance of embryonic radial glial cells in an undifferentiated state, and its downregulation drives the correct maturation and migration of cerebellar and cortical neurons during development (Liu et al., 2019; Singh et al., 2016; Wang et al., 2019). Zeb1 null mice die perinatally with severe skeletal and limb defects, craniofacial abnormalities, as well as respiratory failure and T cell deficiency (Takagi et al., 1998). The lethal phenotype of the Zeb1 null mouse precluded functional studies of ZEB1 in the adult brain. However, we have recently generated a conditional *Zeb1* knockout model that has enabled investigating ZEB1 functions beyond early development (Brabletz et al., 2017).

Here, we used the tamoxifen (TAM)-inducible GLAST::CreER^{T2} model to investigate the effects of *Zeb1* deletion in adult

This is an open access article under the CC BY license (http://creativecommons.org/licenses/by/4.0/).

hippocampal neurogenesis (Brabletz et al., 2017; Madisen et al., 2010; Mori et al., 2006). ZEB1 is expressed in RGL cells and IPCs, as well as mature astrocytes, but is downregulated in the neuronal lineage. We found that ZEB1 is necessary for the maintenance of activated RGL cells; loss of *Zeb1* led to a differentiation-coupled depletion of the RGL pool accompanied by a transient increase in neurogenesis and a loss of SGZ-derived astrocytes. In addition to increased neuronal production, we found increased survival of neurons during their maturation. Analysis of individual RGL cell clones showed that most clones in *Zeb1*-deleted animals contained only two neurons and no active RGL cell, whereas clones in control mice contained active RGL cells and a mixture of neurons and astrocytes. Further analysis of mitotic figures *in vivo* and time-lapse imaging *in vitro* revealed that *Zeb1* deletion increased symmetrical divisions in comparison with control mice, leading to precocious pro-neuronal differentiation. Mechanistically, we identify ZEB1 as a transcriptional activator of the glial lineage transcription factor ETV5, and targeted expression of ETV5 increased asymmetrical divisions and decreased neuronal differentiation. Together, our data show that ZEB1 is necessary for self-renewal of hippocampal RGL cells by promoting asymmetrical cell divisions and inducing expression of ETV5.

RESULTS

ZEB1 is a known regulator of stemness in many tissues (Goossens et al., 2017). Recent studies showed that ZEB1 functions in embryonic neurogenesis (Liu et al., 2019; Singh et al., 2016; Wang et al., 2019), and we previously found that ZEB1 is crucial for the self-renewal of glioblastoma cancer stem cells (Hoang-Minh et al., 2018; Jimenez-Pascual et al., 2019; Siebzehnrubl et al., 2013). Hypothesizing that ZEB1 would execute similar functions in adult neural stem cells, we evaluated the consequences of conditional-inducible Zeb1 deletion in the adult hippocampus as a paradigm of a well-characterized neurogenic niche.

ZEB1 is expressed in hippocampal stem and progenitor cells and astrocytes

We assessed co-expression of ZEB1 with cell-type-specific markers in the adult hippocampal DG (Figure 1A) of 12-week-old mouse brain tissue sections. Co-staining for ZEB1 and glial fibrillary acidic protein (GFAP; Figure 1B) showed that ZEB1 was abundantly expressed in RGL cells (Figure 1B′) and in mature astrocytes in the hilus (Figure 1B″). ZEB1 is present in virtually all SOX9$^+$ astrocytes within the DG (Figure 1C). We next quantified ZEB1 in quiescent (GFAP$^+$MCM2$^-$) and activated (GFAP$^+$MCM2$^+$) RGL cells, as well as in IPCs (GFAP$^-$MCM2$^+$). This revealed that ZEB1 expression is strongly associated with MCM2$^+$ cells, while quiescent RGL cells are mostly ZEB1 negative (Figure 1D). Quantification of ZEB1$^+$ RGL cells confirmed this, showing that ZEB1 overwhelmingly labels active RGL cells (Figure 1E). ZEB1 was absent within the neuronal lineage, including Doublecortin-positive (DCX$^+$) early neuronal cells (Brown et al., 2003) and NeuN$^+$ mature neurons (Figures 1F and 1G). Hence ZEB1 is expressed in active RGL cells (GFAP$^+$MCM2$^+$), IPCs (GFAP$^-$MCM2$^+$), and astrocytes but is downregulated once cells undergo neuronal lineage commitment. This is supported by published datasets from single-cell RNA sequencing studies (Figure S1) (Hochgerner et al., 2018). This expression pattern supports a functional role for ZEB1 in adult neural stem and progenitor cells. Interestingly, the continued expression of ZEB1 in astrocytes also suggests a more general role for glial identity, comparable with, e.g., SOX2 (Bani-Yaghoub et al., 2006). Because ZEB1 is absent in quiescent RGL cells and expressed when these become activated, we chose to focus on the functions of ZEB1 in RGL cells.

Mouse model for conditional-inducible *Zeb1* deletion in RGL cells

To evaluate the function of ZEB1 in RGL cells, we generated a conditional-inducible mouse model for deletion of *Zeb1* by crossing the *Zeb1*$^{f/f}$ mouse line (Brabletz et al., 2017) with the TAM-inducible GLAST::CreER^{T2} (Mori et al., 2006) and the Rosa26-tdTomato reporter (Madisen et al., 2010) transgenic lines (Figures 2A and 2B). This model, hereafter referred to as *Zeb1*$^{-/-}$, enabled the deletion of *Zeb1* in neural stem cells and the astroglial lineage combined with lineage tracing. As controls, we used GLAST::CreER^{T2}/Rosa26-tdTomato mice with wild-type levels of ZEB1 expression (hereafter referred to as control). We tested the recombination efficiency 1 day after the last TAM injection. Recombination occurred at a high level and to a comparable extent in both models (Figures 2C and 2D).

Next, we quantified the numbers of ZEB1$^+$ and ZEB1$^-$ cells in GFAP$^+$tdTOM$^+$ cells at 1 day post-induction to determine the efficiency of Zeb1 deletion in RGL cells. There was a 17-fold decrease in ZEB1$^+$ RGL cells following TAM administration in *Zeb1*$^{-/-}$ mice compared with controls (Figures 2E and 2F). This validates successful and efficient knockout of *Zeb1* in RGL cells following TAM administration.

Comparison of control and *Zeb1*$^{-/-}$ mice with compound heterozygotes at 4 weeks post-induction demonstrated that the phenotype of *Zeb1*$^{+/-}$ is not significantly different from the controls (Figure 2G). Hence *Zeb1*$^{-/-}$ mice can be used to ablate *Zeb1* in DG RGL cells, and bi-allelic deletion of *Zeb1* is needed to obtain a significant phenotype.

Zeb1 loss causes depletion of hippocampal RGL cells

Having established that ZEB1 is absent in *Zeb1*$^{-/-}$ mice immediately after TAM administration, we investigated the longer-term effects of *Zeb1* deletion in hippocampal RGL cells. We quantified quiescent (GFAP$^+$MCM2$^-$; Figure 3, arrowheads) and activated (GFAP$^+$MCM2$^+$; Figure 3, arrows) RGL cells at 1 day (Figure 3A) and 4 weeks (Figure 3B) after *Zeb1* deletion. The number of quiescent RGL cells was comparable between control and *Zeb1*$^{-/-}$ mice immediately after induction but was significantly lower by 4 weeks (Figure 3C). By contrast, activated RGL cells showed an immediate decrement at 1 day that was sustained at 4 weeks (Figure 3D). This indicates that *Zeb1* loss causes a steady decline of activated RGL cells, which results in continued recruitment of quiescent RGL cells that exhaust the hippocampal RGL cell pool.

To confirm this, we investigated whether the loss of RGL cells progressed over time, and assessed combined numbers of quiescent and activated RGL cells. *Zeb1*$^{-/-}$ mice continued to

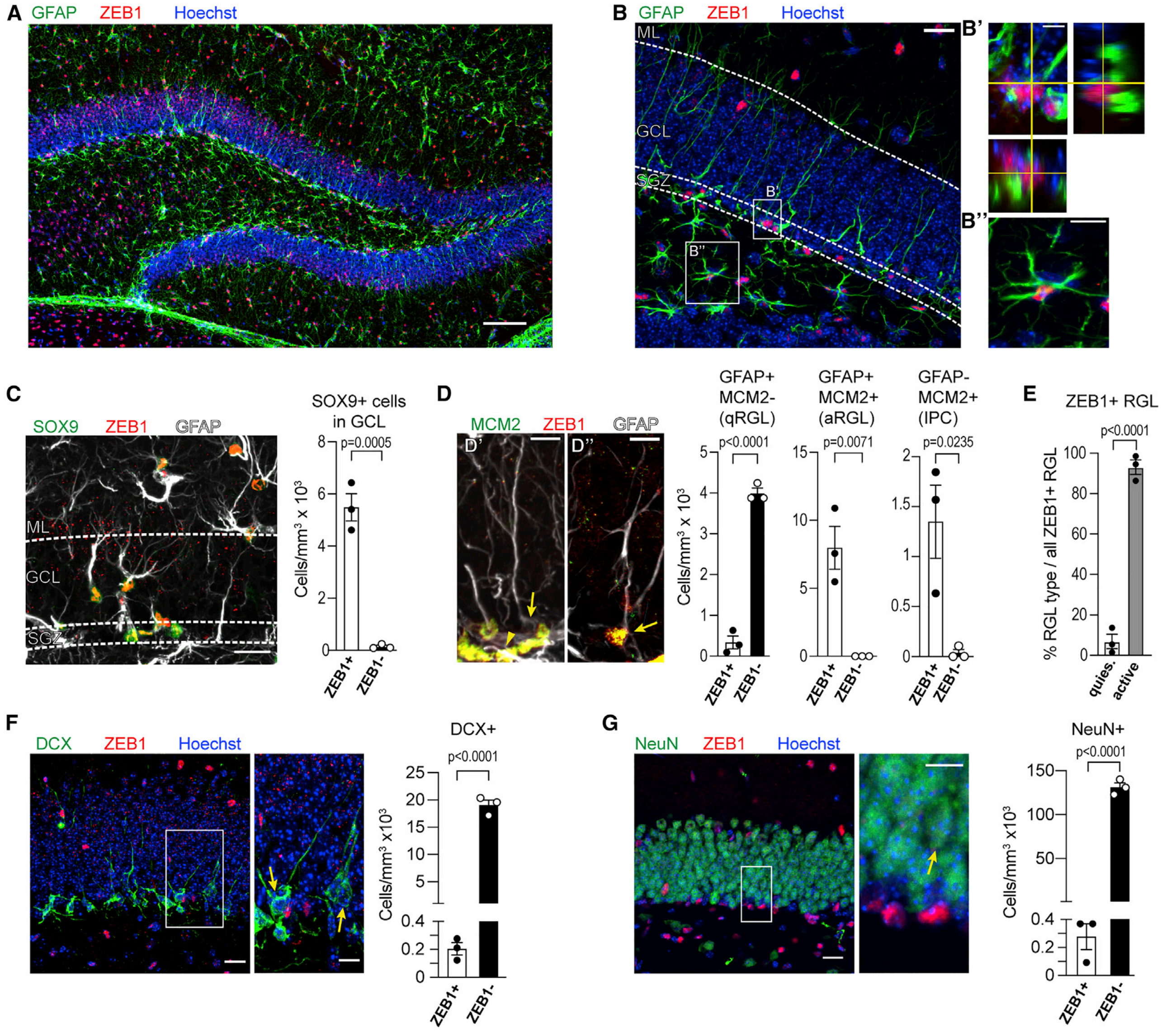

Figure 1. Expression of ZEB1 in the adult mouse hippocampus

(A) Overview of whole DG with co-staining of GFAP and ZEB1.

(B) GFAP and ZEB1 are co-expressed in SGZ RGL cells (B′) and in mature astrocytes (hilus, B″).

(C) SOX9 and ZEB1 are co-expressed in RGL cells and astrocytes within the GCL.

(D) Quiescent (q) RGL cells are mostly negative for ZEB1 (arrow in D′, left bar graph), while the majority of ZEB1$^+$ cells in the SGZ constitute active (a) RGL cells (arrow in D″, middle bar graph) or IPCs (arrowhead in D′, right bar graph).

(E) Fraction of qRGL versus aRGL cells out of all ZEB1$^+$ RGL cells.

(F and G) ZEB1 is absent in DCX$^+$ neuroblasts (F, arrows), as well as in NeuN$^+$ granule neurons (G, arrow).

Dots represent individual mice (minimum of two sections analyzed per animal); numerical data are shown as mean ± SEM. Scale bars, 100 μm (A); 20 μm (B–D, F, and G); 10 μm (insets). GCL, granule cell layer; ML, molecular layer. See also Figure S1.

display decreased RGL cell numbers compared with controls by 8 (Figure 3E) and 12 weeks post-induction (Figure 3F).

The slow-rate depletion of RGL cells suggests an increased rate of differentiation at the expense of self-renewal in this cell population. We therefore sought to determine whether *Zeb1* loss resulted in altered numbers of hippocampal IPCs. Evaluating the number of GFAP$^-$ MCM2$^+$ IPCs revealed a significant increase in *Zeb1*$^{-/-}$ mice at 1 day post-induction but a significant decrease at 4 weeks (Figure 3G). We further quantified TBR2$^+$tdTOM$^+$ IPCs (Figure 3H) in control and *Zeb1*$^{-/-}$ mice between 2 and 12 weeks after *Zeb1* deletion (Figure 3I). TBR2$^+$ IPC numbers in *Zeb1*$^{-/-}$ mice were significantly greater at 2 weeks after induction than in controls, but by 4 weeks, IPC numbers were comparable in both groups. By 12 weeks post-recombination, this effect was inverted, and the numbers of TBR2$^+$ cells in *Zeb1*$^{-/-}$ mice were significantly lower than in controls.

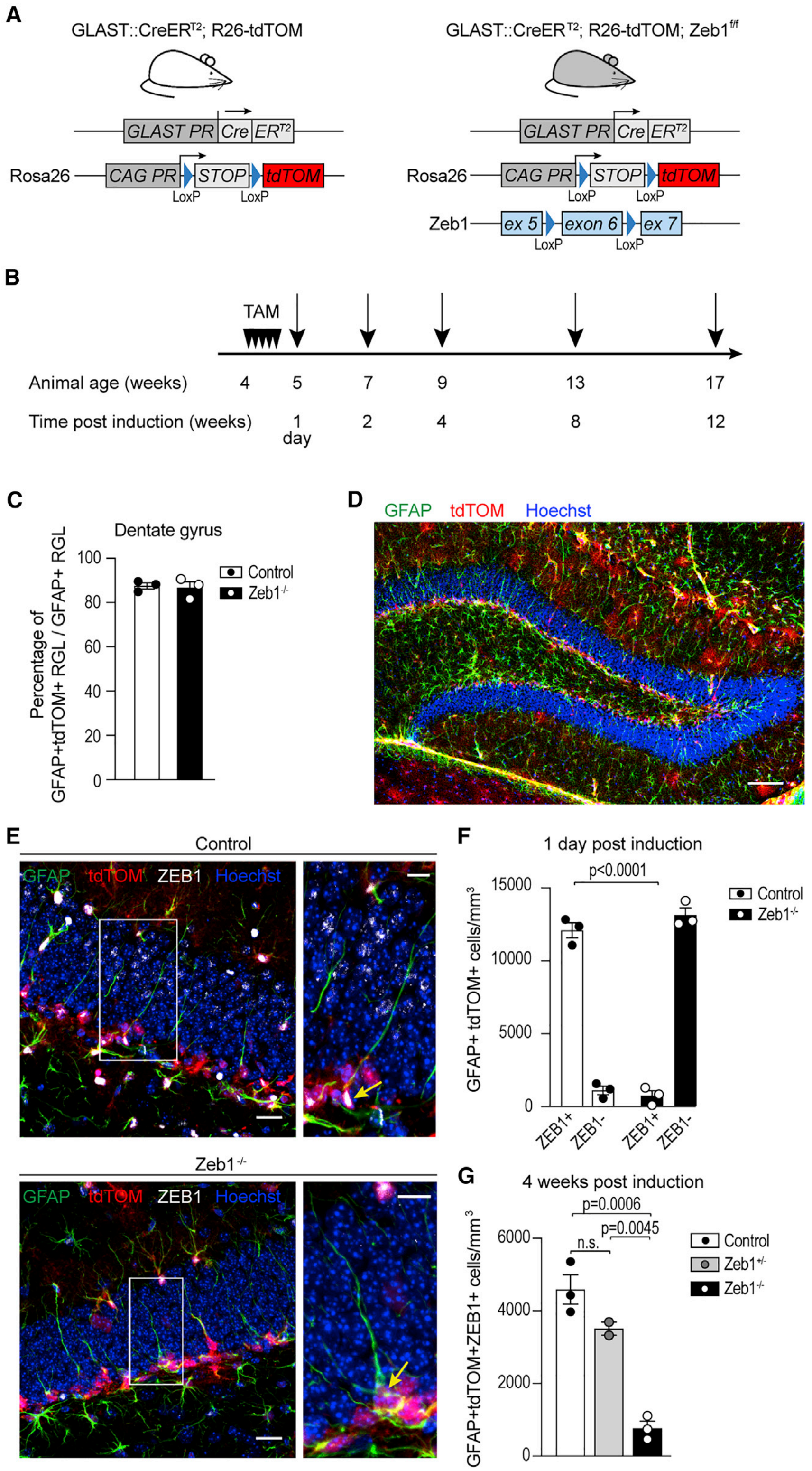

Figure 2. Validation of the conditional-inducible Zeb1 knockout model

(A) Breeding strategy to generate inducible control and Zeb1 knockout mice with endogenous tdTOM reporter expression.

(B) Tamoxifen (TAM) was administered daily for 5 consecutive days. Tissue was harvested at the indicated time points post-induction (black arrows).

(C) Induction of tdTOM in GFAP$^+$ RGL cells was comparable in both models.

(D) Representative image depicting overlap of tdTOM in GFAP$^+$ RGL cells within the SGZ.

(E) Representative images showing RGL cells are ZEB1$^+$ in control mice (arrow), but ZEB1$^-$ following TAM administration in *Zeb1*$^{-/-}$ mice (arrow).

(F) Quantification of ZEB1 expression in GFAP$^+$tdTOM$^+$ RGL cells in the SGZ of control and *Zeb1*$^{-/-}$ mice 1 day post-induction.

(G) Comparison of ZEB1-expressing RGL cells at 4 weeks post-induction in control, *Zeb1*$^{+/-}$, and *Zeb1*$^{-/-}$ mice.

Dots represent individual mice (minimum of two sections analyzed per animal); numerical data are shown as mean ± SEM. Scale bars: 100 μm (D); 20 μm (F); 10 μm (insets).

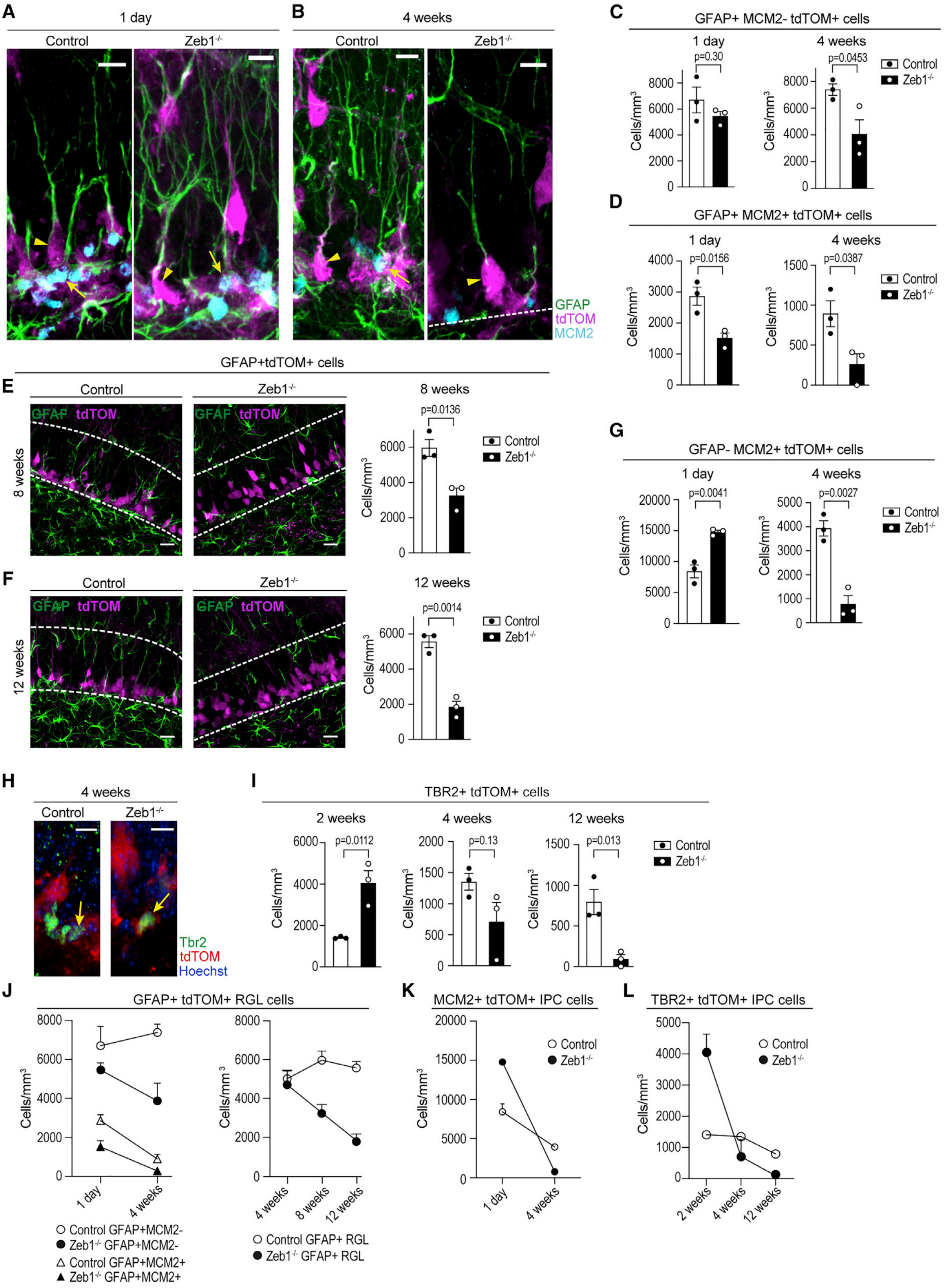

(legend on next page)

Direct comparison of the trajectories of quiescent, activated, and total RGL cells over time highlights the steady decline of RGL cells in *Zeb1*$^{-/-}$ mice, while this population remains at equilibrium in controls (Figure 3J). The increased differentiation of RGL cells following *Zeb1* loss results in a transient increase of IPCs that is reverted as RGL cell numbers are depleted (summarized in Figures 3K and 3L).

Loss of *Zeb1* increases neurogenesis in the hippocampus

We determined whether the transient IPC increase in *Zeb1*$^{-/-}$ mice translated into increased numbers of differentiated progenies. To investigate the fate of the progenitor cells generated through the division-coupled depletion of RGL cells, we assessed the numbers of neuroblasts and neurons in the DG.

We first quantified newly generated neuroblasts and found that the trajectory of DCX^{+}tdTOM^{+} cells in the DG of *Zeb1*$^{-/-}$ mice followed a similar pattern as IPCs. Compared with controls, the numbers of DCX^{+} cells in *Zeb1*$^{-/-}$ mice were significantly greater at 2 and 4 weeks post-induction. However, this distribution inverted at 8 weeks when the number of DCX^{+} cells was significantly lower in *Zeb1*$^{-/-}$ mice compared with controls, in line with the observed decline in RGL cells and IPCs (Figures 4A–4C).

Because of the transient amplification of neuroblasts after *Zeb1* deletion, we probed whether these cells matured into granule neurons. We followed the expected timeline of neuronal maturation and compared NeuN^{+}tdTOM^{+} granule neurons from 4 to 12 weeks post-*Zeb1* deletion. Although we observed no differences in mature neuron numbers at 4 weeks, the numbers of NeuN^{+} neurons were significantly greater at 8 and 12 weeks post-induction in *Zeb1*$^{-/-}$ mice than in controls (Figures 4D–4F). Mature neuron numbers showed a linear increase in control mice but plateaued in *Zeb1*$^{-/-}$ mice between 8 and 12 weeks. Hence *Zeb1* deletion results in a prominent increase in neuronal differentiation.

We next evaluated whether the increase in newborn neurons is linked to proliferation rates of stem/progenitor cells in *Zeb1*$^{-/-}$ mice and quantified co-expression of the proliferation marker Ki67 in GFAP^{+}tdTOM^{+} RGL cells and TBR2^{+}tdTOM^{+} IPCs at 1 day and 2 weeks post-induction. There was no significant difference in proliferating cells between *Zeb1*$^{-/-}$ and control at either time point (Figures S2A–S2E). Additionally, we performed EdU labeling *in vivo* concurrent with TAM administration (Figure S2F) and analyzed numbers of EdU^{+}tdTOM^{+} cells at 2 weeks after labeling. We found that both DCX^{+}EdU^{+}tdTOM^{+} and DCX^{-}EdU^{+}tdTOM^{+} cell numbers were significantly increased in *Zeb1*$^{-/-}$ mice (Figures S2G and S2H). Most newborn neurons generated during neurogenesis undergo apoptosis, and only a small fraction survives and integrates successfully into the hippocampal circuitry (Dayer et al., 2003; Ryu et al., 2016). Changes in neuronal survival may considerably impact the number of neurons generated by neurogenesis (Sierra et al., 2010). Therefore, we determined whether *Zeb1* loss affected survival of neuronal progenies and quantified co-expression of the apoptotic marker cleaved caspase-3 with either DCX (at 2 weeks post-induction) or NeuN (at 4 weeks post-induction). The number of apoptotic DCX^{+} neuroblasts was not significantly different between *Zeb1*-deleted mice and controls, but we did find significantly fewer apoptotic NeuN^{+} granule neurons in the *Zeb1*$^{-/-}$ DG (Figures S2I and S2J). This indicates that *Zeb1* loss promotes long-term survival of newly generated hippocampal neurons, which may contribute to the increased numbers of mature neurons following *Zeb1* deletion.

Zeb1 deletion during embryonic neurogenesis results in increased migration of neuroblasts (Liu et al., 2019; Singh et al., 2016). Neuroblasts in *Zeb1*$^{-/-}$ mice did not migrate differently compared with control mice, as evidenced by measurement of distances between the bottom of the SGZ and the positions of EdU^{+}DCX^{+}tdTOM^{+} cells within the DG (Figure S2K).

To test whether newborn neurons displayed normal maturation, we performed patch-clamp electrophysiology (Figures 4G, 4H, S3A, and S3B). This revealed no significant differences between resting membrane potential, input resistance, membrane time constant, or membrane capacitance in tdTOM^{+} *Zeb1*$^{-/-}$ and control DG granule cells (DGGCs) at 4–5 weeks post-induction. We also found no significant differences in these properties between tdTOM^{-} *Zeb1*$^{-/-}$ and control DGGCs cells, or between tdTOM^{+} and tdTOM^{-} cells of both genotypes (Figure 4H). Furthermore, no differences were observed in cellular excitability, with all four groups of DGGCs having similar action potential firing properties (Figures S3A and S3B). In reconstructed tdTOM^{+} DGGCs, we found no significant differences in dendritic morphology 4–5 weeks post-induction (Figures S3C and S3D). Interestingly, and in contrast with the effects of *Zeb1* deletion in embryos (Liu et al., 2019; Singh et al., 2016; Wang et al., 2019), our findings suggest that *Zeb1* knockout does not markedly alter the functional and morphological development or migration of DGGCs (Figures S2K and S3E–S3G).

Decreased astrocyte numbers in the SGZ, but not DG, of *Zeb1*$^{-/-}$ mice

Zeb1 loss causes depletion of RGL cells by inducing proneuronal differentiation, but RGL cells in the DG also generate

Figure 3. Effects of Zeb1 loss in RGL cells and IPCs

(A and B) Representative images of quiescent (GFAP^{+}MCM2^{-}tdTOM^{+}; arrowheads) and activated (GFAP^{+}MCM2^{+}tdTOM^{+}; arrows) RGL cells in the SGZ of control and *Zeb1*$^{-/-}$ mice at 1 day (A) and 4 weeks (B) post-induction.

(C) Numbers of quiescent RGL cells at 1 day and 4 weeks post-*Zeb1* deletion.

(D) Numbers of activated RGL cells at 1 day and 4 weeks post-*Zeb1* deletion.

(E and F) Representative images and quantification of RGL cells at 8 (E) and 12 weeks (F) post-induction.

(G) Numbers of GFAP-MCM2^{+}tdTOM^{+} IPCs at 1 day and 4 weeks post-*Zeb1* deletion.

(H and I) Representative images at 4 weeks post-induction (H) and quantification of TBR2^{+} IPCs at 2, 4, and 12 weeks post-Zeb1 deletion (I).

(J–L) Summary graphs depict quiescent/activated (left) and total (right) RGL populations in control and *Zeb1*$^{-/-}$ mice (J). Summary graphs outline the temporal changes of GFAP^{-}MCM2^{+} (K) and TBR2^{+} (L) IPCs.

Dots represent individual mice (minimum two sections analyzed per animal), except in (J)–(L), where dots represent averages. Numerical data are shown as mean ± SEM. Dashed lines in images demarcate DG boundaries. Scale bars: 20 μm (insets: 10 μm).

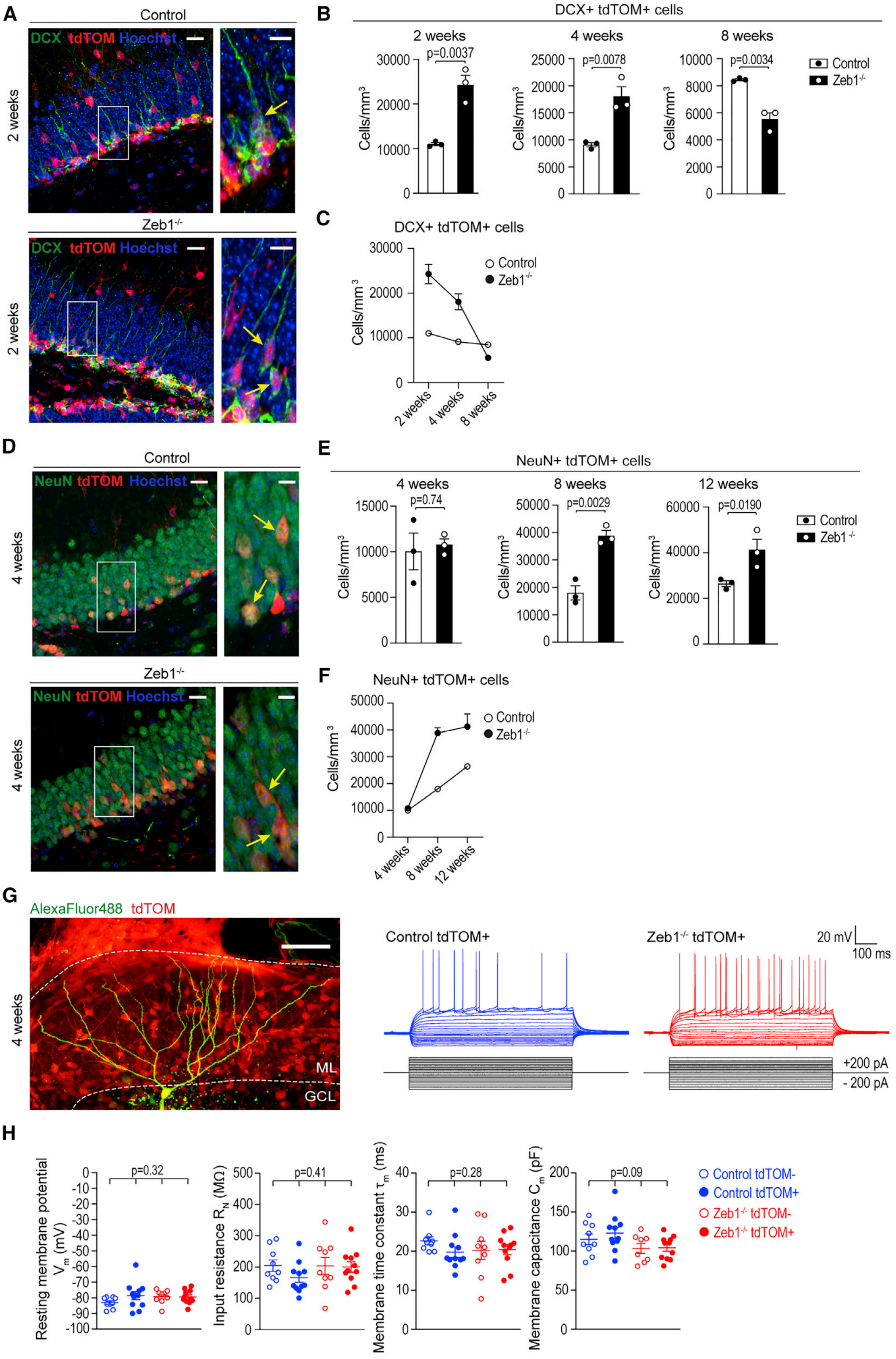

(legend on next page)

astrocytes (Bonaguidi et al., 2011; Encinas et al., 2011; Gebara et al., 2016). Because ZEB1 is present in all hippocampal astrocytes, we evaluated whether the neuronal differentiation-coupled depletion of RGL cells affected production of astrocytes in the SGZ and/or whether *Zeb1* deletion affected astrocyte numbers in general. Because the GLAST promoter is active in both RGL cells and normal astrocytes in control and *ZEB1*$^{-/-}$ mice, we assessed numbers of SOX9$^+$ non-RGL astrocytes (Figure 5A) and S100β$^+$ (Figure 5B) astrocytes separately within the SGZ and the DG. This enabled separating the effects of *Zeb1* deletion on RGL cell astrogliogenesis while excluding confounding effects from *Zeb1* loss in astrocytes in other areas of the DG and the hilus.

In the SGZ of *Zeb1*$^{-/-}$ mice, numbers of SOX9$^+$ astrocytes were significantly decreased at 4 and 8 weeks post-induction, which was matched by numbers of S100β$^+$ astrocytes at 8 and 12 weeks post-induction (Figure 5C). By contrast, we did not observe significant differences in SOX9$^+$ or S100β$^+$ astrocytes in the granule cell layer of the DG at the same time points (Figure 5D). Because ZEB1 affected survival of newborn neurons, we further evaluated whether *Zeb1* loss altered survival of GFAP$^+$tdTOM$^+$ RGL cells at 1 day post-induction and/or SOX9$^+$tdTOM$^+$ non-RGL astrocytes at 4 weeks post-induction. Although overall cell counts of cleaved caspase-3$^+$ cells in both populations were low, we found no difference in apoptotic RGL cells or astrocytes between control and *Zeb1*$^{-/-}$ mice (Figures 5E and 5F). This indicates that ZEB1 may be dispensable for RGL and astrocyte survival.

In summary, our data support that *Zeb1* deletion in hippocampal RGL cells promotes their differentiation into neuronal progenies, while simultaneously preventing astroglial fate specification. Newborn *Zeb1*$^{-/-}$ neurons have a higher survival rate than control neurons and integrate into the DG. In contrast, we found no evidence for decreased survival of astrocytes following *Zeb1* deletion, suggesting that reduced astrocyte numbers may be caused by changes in astrogliogenesis. To further elucidate whether *Zeb1* loss results in diminished production of new astrocytes, we analyzed the cellular composition of individual RGL clones.

Clonal lineage composition is altered in *Zeb1*$^{-/-}$ compared with control mice

To investigate how neurogenesis and astrogliogenesis are altered in *Zeb1*$^{-/-}$ mice, we employed a low-dose induction paradigm that enabled sparse recombination and subsequent lineage tracing in individual RGL cells (Figures 6A, 6B, and S4A–S4E) (Bonaguidi et al., 2011). We compared clonal lineages in control and *Zeb1*$^{-/-}$ mice 4 weeks after recombination and found a significant reduction in active clones (containing an RGL cell and non-RGL progenies), as well as a significant increase in depleted clones (containing only differentiated progenies and no RGL cell), in *Zeb1*$^{-/-}$ mice (Figures 6C, S4D, and S4F). These RGL-depleted clones confirm the previously observed RGL cell loss following *Zeb1* deletion (Figure 3). The frequency of quiescent clones (containing a single RGL cell) was similar in control and *Zeb1*$^{-/-}$ mice (Figures 6C and S4F). This indicates that *Zeb1* loss does not directly affect quiescent RGL cells, but its effects manifest only after RGL cells become activated. This is further supported by the lack of ZEB1 expression in quiescent RGL cells (Figure 1E) and the delayed decline in quiescent RGL cell numbers, whereas activated RGL cell numbers decrease immediately following TAM administration (Figure 3).

Because of the increase in neurogenesis and concomitant decrease in astrocyte numbers in the SGZ, we asked whether *Zeb1* loss may alter the ratio of neuron/astrocyte production. We analyzed clonal lineages from individual RGL cells in active and depleted clones (Bonaguidi et al., 2011). A significantly lower number of clones contained astrocytes in *Zeb1*$^{-/-}$ mice compared with controls (Figure 6D). Although we found a few clones containing only an RGL cell and an astrocyte (RA) in controls, these were absent in *Zeb1*$^{-/-}$ mice. More importantly, approximately 30% of active clones in control mice contained both astroglia and neurons (RAN), whereas we found only a single bi-lineage clone in *Zeb1*$^{-/-}$ animals. There was no significant difference in the frequency of active clones that produce only neurons (RN) between controls and *Zeb1*$^{-/-}$ mice. We next compared the ratio of neuron-only-producing versus bi-lineage (neuron- and astrocyte-producing) clones and found that this ratio is significantly skewed toward neuron-only-producing clones after *Zeb1* loss (Figures 6E, S4G, and S4H). This indicates that ZEB1 is not only relevant for self-renewal but also for lineage selection in hippocampal RGL cells by preventing neuronal and promoting astroglial fate.

To elucidate the capacity for clonal expansion following *Zeb1* deletion, we assessed the number of cells per clone in both groups. When comparing the cell numbers across individual clones, we observed an enrichment of clones containing either two or five cells in control mice, whereas most *Zeb1*$^{-/-}$ clones contained one to three cells (Figure S4I). This is consistent with

Figure 4. Effects of Zeb1 loss in newborn neurons

(A) Representative images of DCX$^+$tdTOM$^+$ neuroblasts (arrows) at 2 weeks post-induction.

(B) Quantification of DCX$^+$tdTOM$^+$ neuroblasts in the DG of control and *Zeb1*$^{-/-}$ mice at 2, 4, and 8 weeks post-induction.

(C) Summary graph of neuroblast changes over time.

(D) Representative images of NeuN$^+$ granule neurons (arrows) at 4 weeks post-induction.

(E) Numbers of NeuN$^+$tdTOM$^+$ granule neurons at 4, 8, and 12 weeks post-induction.

(F) Summary graph showing granule neuron changes over time.

(G) 2D projection of a two-photon image z stack showing a typical granule cell filled with Alexa 488 via the patch-clamp recording electrode. Representative recordings from tdTOM expressing *Zeb1*$^{-/-}$ (red) and control (blue) DGGCs.

(H) Scatterplots show resting membrane potential (R_m), input resistance (R_N), membrane time constant (τ_m), and membrane capacitance (C_m) for individual DGGCs overlaid with the mean for each group.

Additional graphs and Neurolucida traces are in Figure S3. Dots represent individual mice (minimum of two sections analyzed per animal; B and E), average values (C and F), or individual neurons (4 mice/genotype, H). Numerical data are shown as mean ± SEM. Scale bars: 20 μm (insets: 10 μm). See also Figures S2 and S3.

CellPress
OPEN ACCESS

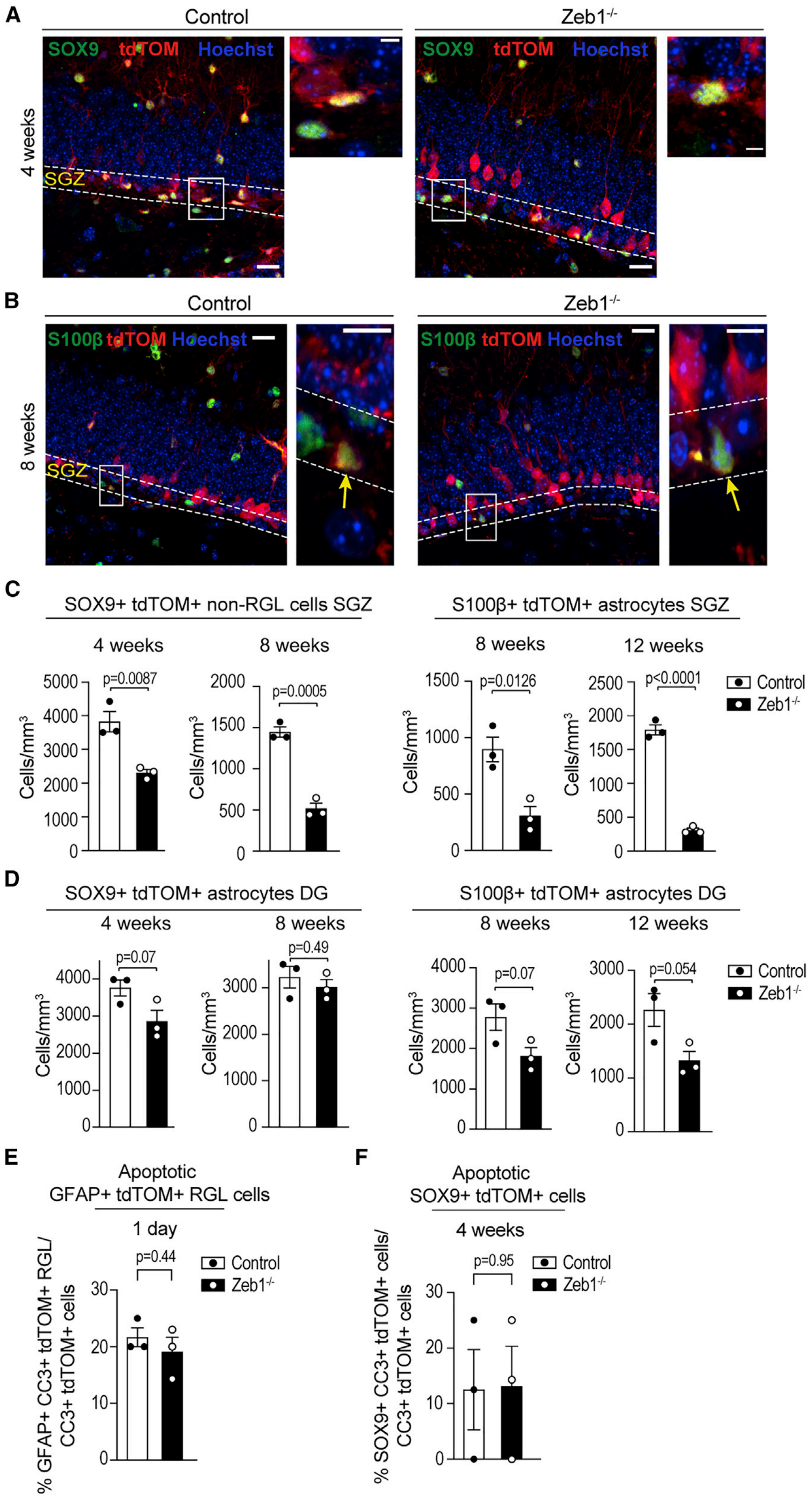

Figure 5. Effects of ZEB1 loss in astrocytes

(A) Representative images at 4 weeks post-induction, identifying SOX9$^+$ SGZ astrocytes (insets).

(B) Representative images at 8 weeks post-induction, identifying S100β$^+$ SGZ astrocytes (arrows).

(C) Quantification of SOX9$^+$tdTOM$^+$ non-RGL astrocytes in the SGZ (left) at 4 and 8 weeks post-induction and of S100β$^+$tdTOM$^+$ SGZ astrocytes (right) at 8 and 12 weeks post-induction.

(D) Quantification of SOX9$^+$tdTOM$^+$ (left) and S100β$^+$tdTOM$^+$ (right) astrocytes in the DG.

(E) Fraction of apoptotic GFAP$^+$ RGL at 1 day post-induction.

(F) Fraction of SOX9$^+$ apoptotic astrocytes at 4 weeks post-induction.

Dots represent individual mice (minimum of two sections analyzed per animal); numerical data are shown as mean ± SEM. Scale bars: 20 μm (insets: 10 μm).

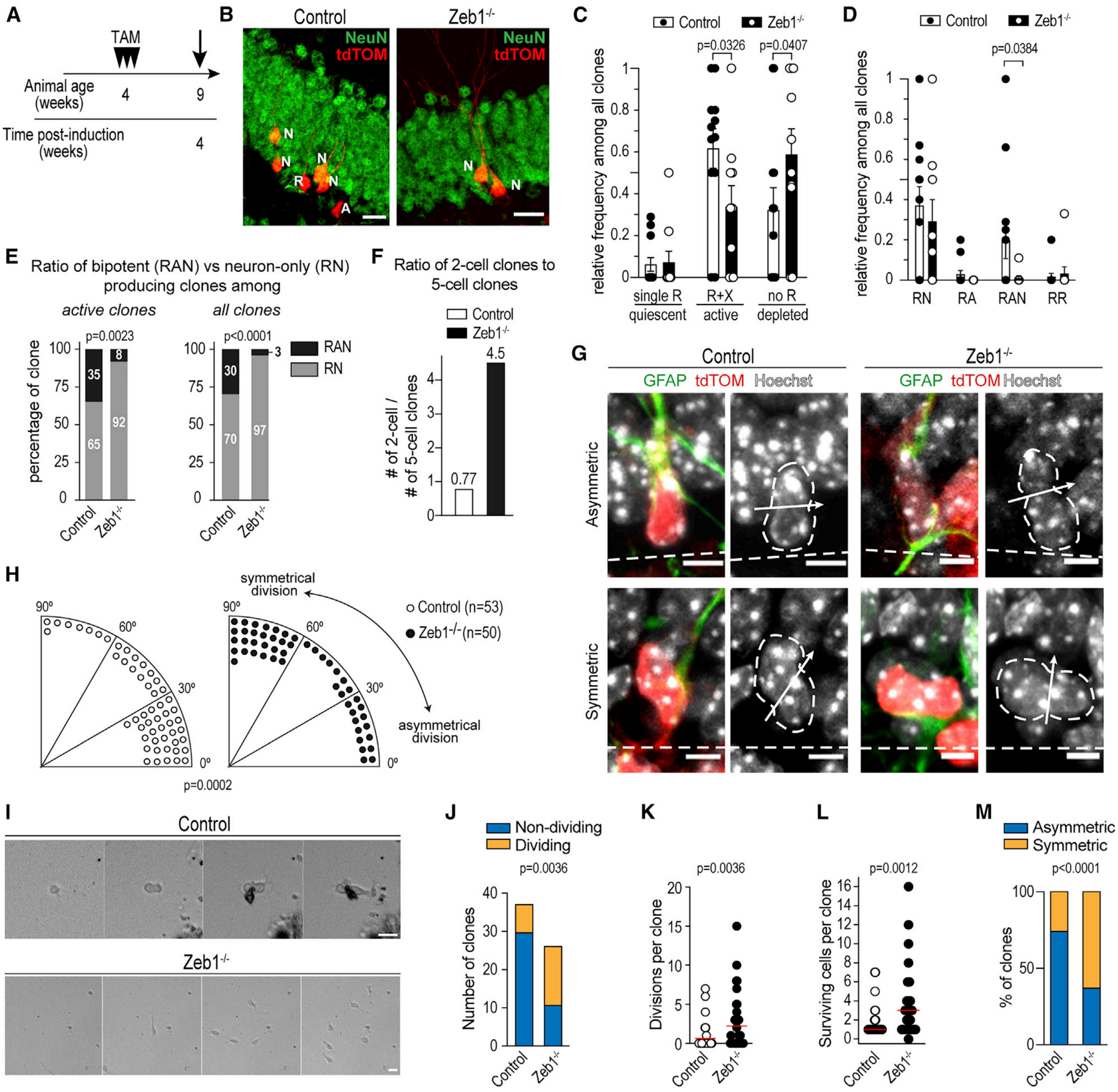

Figure 6. Analysis of RGL cell clones

(A) Mice were injected with low-dose TAM (0.05 mg), and recombination was assessed 4 weeks post-induction.

(B) Representative images of clones at 4 weeks post-induction.

(C) Relative frequency of quiescent (containing only an RGL cell [single R]), active (containing an RGL cell and any other cell type [R+X]), and depleted (containing only lineage-restricted cells [no R]) clones (n = 38 [control, from 12 hippocampi] versus 35 [*Zeb1*$^{-/-}$, from 10 hippocampi]).

(D) Frequencies of active clone subtypes (relative to all clones; n = 26 [control] versus 14 [*Zeb1*$^{-/-}$]). Clone subtypes are neurogenic (RGL cell and neurons [RN]), astrogliogenic (RGL cell and astrocyte [RA]), bi-lineage (RGL cell, neuron(s) and astrocyte [RAN]), or self-renewing (two RGL cells [RR]).

(E) Frequencies of bi-lineage (RAN) versus neuron-only-producing (RN) clones across active clones (containing RGL cell; left) and all clones (right).

(F) Ratio of clones containing two cells versus clones containing five cells.

(G) Representative images of cleavage plane orientation in RGL cells undergoing asymmetric (top) or symmetric (bottom) division in control and *Zeb1*$^{-/-}$ mice. Dashed lines indicate SGZ-hilus border.

(H) Quantification of RGL cell division angles, binned into 30° groups.

(I) Representative images from *in vitro* time-lapse imaging of primary adult hippocampal cells.

(J) Quantification of dividing versus non-dividing clones.

(K) Numbers of cell divisions per clone.

(legend continued on next page)

a premature differentiation of RGL cells in *Zeb1*$^{-/-}$ mice, which prohibited further clonal expansion. In support, most neuron-containing clones were active in control mice (Figure S4J), whereas in *Zeb1*$^{-/-}$ mice, neuron-containing clones were mostly depleted (Figure S4K). Premature differentiation of RGL cells after *Zeb1* loss should result in an increased frequency of smaller clones. Therefore, we analyzed the ratio of clones containing two cells versus clones containing five cells and found that this is close to 1 in controls (i.e., similar numbers of clones contain two or five cells), but two-cell clones were 6-fold enriched in *Zeb1*$^{-/-}$ mice (Figure 6F). This further supports that *Zeb1* deletion causes loss of RGL cells as a result of premature differentiation.

We noted that this preferential production of two-cell clones that lack an RGL cell but contain two neurons (Figure S4L) is suggestive of symmetrical cell division that causes differentiation of the mother RGL cell. Therefore, we measured cleavage plane orientation of dividing tdTOM^{+}GFAP^{+} RGL cells relative to their orientation along the SGZ-hilus border in control and *Zeb1*$^{-/-}$ mice (Figures 6G and 6H). We grouped cleavage plane angles into 30° bins and found that *Zeb1* loss results in a significant shift in cell division angles. Although in control animals most RGL cell divisions occurred along the horizontal axis (i.e., asymmetrical) (Falk et al., 2017), the division plane was mostly vertical in *Zeb1*$^{-/-}$ mice. This indicates that *Zeb1*$^{-/-}$ RGL cells are more likely to undergo symmetrical division and, taken together with the greater probability for *Zeb1*$^{-/-}$ clones to contain two neurons, supports that these symmetrical divisions are neurogenic and cause depletion of the RGL cell.

To validate changes in cell division type following *Zeb1* loss, we isolated primary hippocampal cells from 6- to 8-week-old mice and performed *in vitro* time-lapse imaging of individual clones (Figure 6I). *Zeb1*$^{-/-}$ clones were more likely to divide (Figures 6J and 6K), and they produced more surviving cells per clone (Figure 6L). This is in line with our observations on increased cell production and survival following *Zeb1* loss (Figures 3, 4, and S2). Importantly, we found significantly more *Zeb1*$^{-/-}$ clones undergoing symmetric divisions compared with controls, which were more likely to divide asymmetrically (Figure 6M). ZEB1 deficiency causes diminished proliferation of radial glia in the ventricular zone of the developing embryo (Liu et al., 2019). Thus, it is conceivable that RGL cells may be dividing slower after Zeb1 deletion, contributing to the smaller clonal size observed in the former group. Although *in vivo* quantification did not show significant differences of Ki67^{+} RGL cells, we observed a significant delay between the first and second cell division in *Zeb1*$^{-/-}$ clones *in vitro* (Figure S4M). Subsequent cell division timings were not different between control and *Zeb1*$^{-/-}$ clones, indicating that this effect may be transient. In summary, *in vivo* and *in vitro* data support that increased symmetrical divisions underlie the differentiation-coupled depletion of *Zeb1*$^{-/-}$ RGL cells.

Zeb1 regulates expression of the glial lineage transcription factor Etv5

To elucidate how ZEB1 affects cell division planes and fate specification, we curated a list of candidate regulators of asymmetrical cell division. We used the 21 genes listed in the Gene Ontology term for asymmetrical division (GO:0008356). Next, we removed candidates where publicly available chromatin immunoprecipitation (ChIP) sequencing (ChIP-seq) data in glioblastoma cells indicated no promoter occupancy by ZEB1 (Rosmaninho et al., 2018). We then considered candidates expressed in hippocampal RGL cells and astrocytes based on published transcriptional profiling datasets (Chai et al., 2017; Hochgerner et al., 2018). Finally, we selected candidates based on their co-expression with ZEB1 in astrocytes using single-cell RNA-sequencing data (Zeisel et al., 2015) (Table S1; Figure 7A). We validated the resulting shortlist of candidates (ARHGEF2, ETV5, INSC, PARD3, SOX5, and RAB10) by ChIP-PCR to test their promoter occupancy by ZEB1. This yielded positive results for *Arhgef2*, *Etv5*, *Insc*, and *Rab10* (Figures 7B, 7C, and S5A–S5C), and we next determined whether expression of these candidates changed after *Zeb1* deletion. Although we did not observe noticeable differences in ARHGEF2, INSC, or RAB10 levels (data not shown), ETV5 protein levels were significantly lower in primary hippocampal neurosphere cultures from *Zeb1*$^{-/-}$ mice compared with controls (Figure 7D). Importantly, ETV5 is a transcription factor that promotes glial fate specification and regulates branching morphogenesis, a developmental process dependent on changing the cell division plane (Ahmad et al., 2019; Breunig et al., 2015; Chotteau-Lelievre et al., 2003).

We performed immunostaining for ETV5 in hippocampal tissue sections and observed ETV5 in RGL cells of control, but not *Zeb1*$^{-/-}$, mice (Figure 7E). Quantification of ETV5^{+} cells in control animals showed that the majority of RGL cells express ETV5 (Figure 7F). We also identified a subset of ETV5^{+} cells that were non-RGL cells in the SGZ (Figure S5D). Quantification of ETV5^{+} RGL (GFAP^{+}tdTOM^{+}) cells revealed a significant decrease of total ETV5^{+} RGL cell numbers (Figure 7G) and the percentage of RGL cells expressing ETV5 (Figure 7H) in *Zeb1*$^{-/-}$ mice at 1 day post-induction. This demonstrates that *Zeb1* loss causes decreased expression of ETV5 in RGL cells.

To further validate the role of ETV5 in asymmetric division and cell fate, we performed *in vitro* time-lapse imaging of primary hippocampal *Zeb1*$^{-/-}$ cells after transduction with a lentiviral vector containing an ETV5 expression cassette (Figure 7I). We found that ETV5 overexpression restored the ratio of asymmetric to symmetric divisions in *Zeb1*$^{-/-}$ clones *in vitro* (Figure 7J) and significantly reduced cell division times (Figure S5E). Moreover, ETV5 overexpression significantly decreased the number of DCX^{+} progenies from *Zeb1*$^{-/-}$ clones (Figure 7K).

Together, our findings show that (1) ZEB1 binds directly to the *Etv5* promoter in hippocampal neurospheres and induces ETV5 expression, (2) loss of *Zeb1* leads to reduction of ETV5

(L) Numbers of surviving cells per clone.
(M) Ratio of symmetric to asymmetric divisions across all clones.
Dots represent individual clones from 6–7 mice/genotype (C and D), individual cells from 7–8 mice/genotype (H), and individual cells from 5–6 mice/genotype (K and L). Numerical data shown as mean ± SEM. Red line in (K) and (L) represents median. Scale bars: 10 μm (B and G); 20 μm (I). A, astrocyte; N, neuron; R, RGL cell. See also Figure S4.

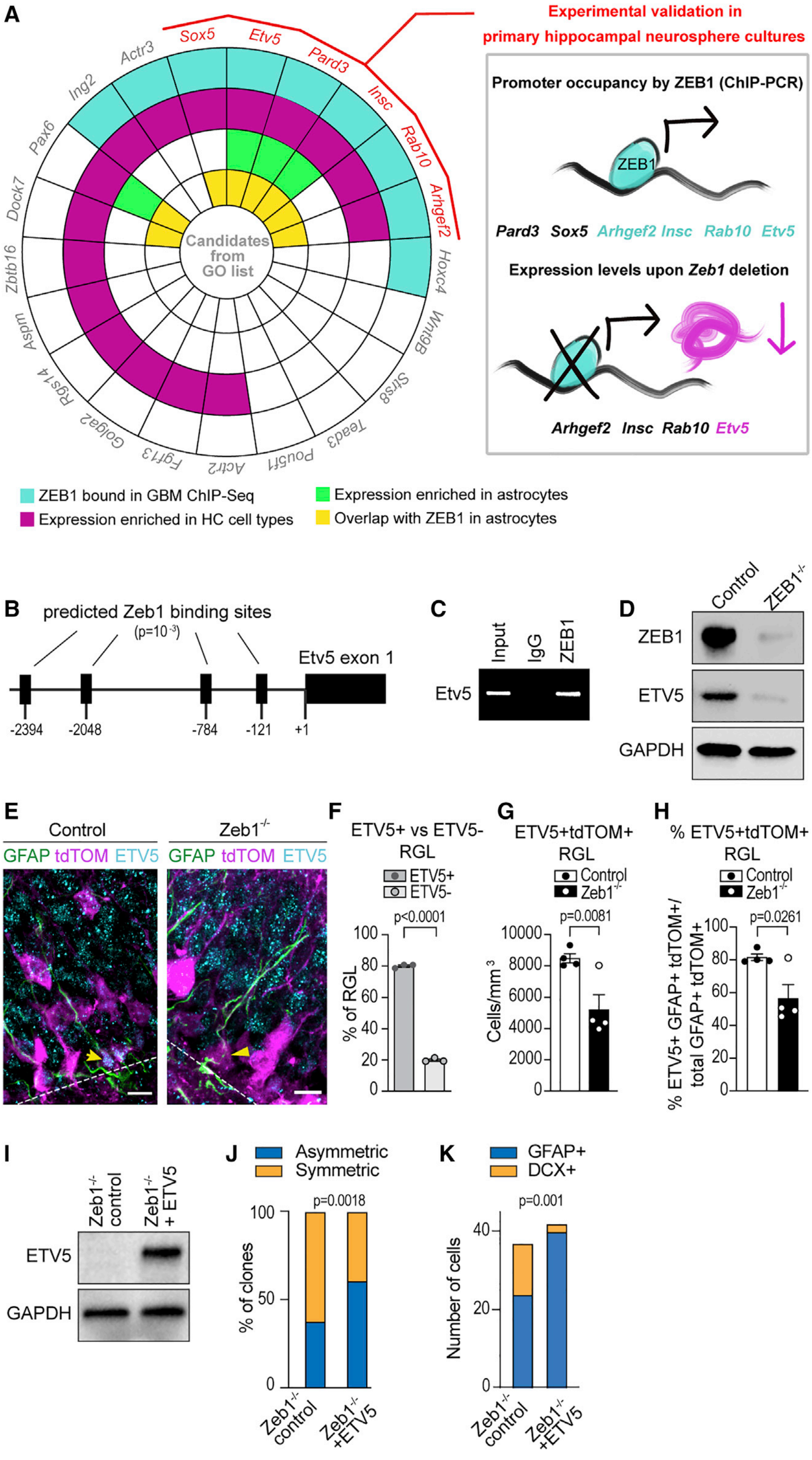

Figure 7. ZEB1 directly regulates expression of ETV5

(A) Workflow for narrowing down the list of candidates relevant for asymmetric division.

(B) Predicted ZEB1 binding sites with a p value of 10^{-3} (based on the JASPAR database; Fornes et al., 2020) in the *Etv5* promoter region.

(C) ChIP of the *Etv5* promoter after pulldown with ZEB1 from hippocampal neurosphere cultures.

(D) Western blot of ETV5 and ZEB1 in hippocampal *Zeb1*$^{-/-}$ and control neurospheres.

(E) Immunofluorescence staining for ETV5 in RGL cells of control and *Zeb1*$^{-/-}$ mice.

(F) Quantification of ETV5 expression in control RGL cells.

(G) Quantification of ETV5$^+$GFAP$^+$tdTOM$^+$ cells with RGL morphology in the SGZ at 1 day post-induction.

(H) Percentage of ETV5$^+$GFAP$^+$tdTOM$^+$ RGL cells out of total GFAP$^+$tdTOM$^+$ RGL cells at 1 day post-induction.

(I) Western blot of ETV5 in hippocampal *Zeb1*$^{-/-}$ neurospheres transduced with a lentiviral ETV5 expression vector and control *Zeb1*$^{-/-}$ cultures.

(J) Ratio of asymmetric to symmetric divisions quantified from time-lapse imaging of primary adult hippocampal cells.

(K) Quantification of GFAP$^+$ and DCX$^+$ cells after live-cell imaging.

Dots represent individual mice (minimum of two sections analyzed per animal); numerical data are shown as mean ± SEM. Scale bars: 10 μm. GBM, glioblastoma; HC, hippocampus. See also Figure S5 and Table S1.

expression in RGL cells, and (3) targeted expression of ETV5 increases asymmetrical divisions in *Zeb1*$^{-/-}$ primary hippocampal cell clones. The lower numbers of DCX$^+$ cells in ETV5-overexpressing clones further indicate that ETV5 expression is negatively correlated with neuronal fate.

DISCUSSION

ZEB1 is a transcriptional regulator of EMT (Peinado et al., 2007). Although primary EMT is essential for cell-state transitions in development, e.g., during neural crest formation (Acloque et al., 2009), the relevance of this process during homeostasis of non-epithelial tissues, such as the adult brain, remains unclear. More recently, the role of EMT-associated transcription factors in the maintenance of stem cell phenotypes has come into focus (Goossens et al., 2017). ZEB1 promotes cancer stemness in glioblastoma (Rosmaninho et al., 2018; Siebzehnrubl et al., 2013), where it is part of an autoregulatory loop together with SOX2 and OLIG2 (Singh et al., 2017), two transcription factors with well-established functions in neural stem/progenitor cells (Ligon et al., 2007; Suh et al., 2007). Here, we investigated the consequences of *Zeb1* deletion in

adult neural stem/progenitor cells. Conditional-inducible deletion of *Zeb1* resulted in rapid and sustained loss of ZEB1 within the DG that was apparent as early as 1 day following TAM administration.

Constitutive deletion of *Zeb1* is lethal around birth, limiting investigations of *Zeb1* deletion to embryonic development. Two studies have reported that downregulation of ZEB1 expression is necessary for the neuronal commitment of precursor cells, allowing them to gain a neuronal identity while migrating to their maturation destinations in the developing cerebellum and cortex, respectively (Singh et al., 2016; Wang et al., 2019). This is partially mirrored in the adult hippocampus because we observed a lack of ZEB1 in the neuroblast and granule neuron populations. Consequently, *Zeb1* deletion results in increased neurogenesis in both embryo and post-natal development. In embryonic neurodevelopment, *Zeb1* loss causes aberrant neuronal morphology and positioning (Liu et al., 2019; Singh et al., 2016; Wang et al., 2019). By contrast, *Zeb1* deletion does not affect morphology, electrophysiological properties, or migration of adult-born granule neurons in the hippocampus. It is possible that adult hippocampal neurogenesis differs from cortical neurogenesis, and that hippocampal granule neurons do not need ZEB1 to mature. Alternatively, migration distances in the adult hippocampus may be too short for a noticeable effect on cell migration.

Another important study investigated ZEB1 functions in embryonic spinal cord glial precursor cells, showing that *Zeb1* loss delays astroglial differentiation (Ohayon et al., 2016). *Zeb1* deletion did not affect specification, proliferation, or survival of astrocyte progenitors in the ventricular zone, but delayed delamination of astroglial precursor cells, which perturbed astroglial differentiation. We report that *Zeb1* loss does not affect survival of astrocytes or RGL cells in the adult DG. Although we find no difference in precursor cell proliferation *in vivo*, time-lapse imaging supports a delay in initial cell divisions *in vitro*. Quantification of astrocyte numbers up to 12 weeks following *Zeb1* deletion did not support delayed astrocytic differentiation in the adult hippocampus. It is conceivable that astrocytic differentiation is delayed beyond the time points studied here, but our data are more consistent with neuronal differentiation-coupled depletion of RGL cells that may also underlie the decrease in astrogliogenesis. Due to low numbers of astrocyte production, we could not test whether ZEB1 is important for migration of newborn astrocytes in the adult hippocampus. It would be interesting to investigate if ZEB1 regulates glial motility in the postnatal or adult brain in future studies.

RGL cells constitute resident stem cells of the DG and generate both neurons and astrocytes throughout life (Bonaguidi et al., 2011; Encinas et al., 2011; Palmer et al., 1997; Seri et al., 2001). Our findings suggest that ZEB1 may be used to identify active RGL cells in combination with GFAP. We report that ZEB1 is necessary for maintenance of activated RGL cells, with *Zeb1* deletion causing a steady loss of activated RGL cells that is coupled with pro-neuronal differentiation (Gao et al., 2011). This differentiation is linked to cell division, because most RGL cell-depleted clones contained more than one neuron, indicating that RGL cells divided at least once prior to depletion. Because quiescent RGL cells do not express ZEB1, the delayed decline of quiescent RGL cell numbers after *Zeb1* deletion is most likely caused by recruitment of quiescent RGL cells to replenish differentiating activated RGL cells. Of note, gene expression datasets from a recent study investigating hippocampal RGL cell maintenance show that ZEB1 levels differ significantly between dormant and resting, but not between resting and active, RGL cells (Harris et al., 2021).

During adult neurogenesis, quiescent RGL cells become activated to generate new neurons. Although some questions remain, there is consensus that RGL cells undergo a limited number of divisions (most likely three) after which they either revert to quiescence or terminally differentiate (Bonaguidi et al., 2011; Encinas et al., 2011; Lazutkin et al., 2019). RGL cells are more likely to differentiate in younger animals, but more frequently they re-enter quiescence in older mice (Harris et al., 2021). In line with these observations and the "disposable stem cell model" (Encinas et al., 2011), we find a 1:3 ratio of RGL cell-depleted to RGL cell-containing clones in control mice, suggesting that over the 4-week chase period, 1 out of 4 clones differentiated. This process is exacerbated after *Zeb1* deletion, increasing the ratio of RGL-depleted to RGL-containing clones (1:1). It would be interesting to test whether *Zeb1* deletion in older mice affects RGL cell return to quiescence (Harris et al., 2021). Most two-cell clones in the *Zeb1*-deficient DG contained only two neurons and no RGL cell, whereas two-cell clones in controls predominantly contained one RGL and one non-RGL cell. Most clones in control mice contained five cells, similar to a live-cell imaging study that tracked neurogenesis *in vivo* (Pilz et al., 2018). It is possible that cell death affected the number of clonal progenies, although we did not find evidence for increased apoptosis at any time point. The higher ratio of two-cell to five-cell clones and the exclusively neuronal content of most two-cell clones in *Zeb1*$^{-/-}$ mice suggest that *Zeb1*$^{-/-}$ RGL cells are more likely to differentiate upon division. Interestingly, time-lapse imaging revealed increased cell division times during initial mitoses after *Zeb1* loss, which may indicate that defunct cell divisions underlie a division-coupled differentiation of RGL cells. Thus, ZEB1 acts to maintain activated RGL cells and prevent their premature differentiation.

Zeb1 loss increased neurogenesis in the adult hippocampus, comparable with genetic deletion of other stem cell transcription factors. For instance, deletion of RBPJκ, the main effector of Notch signaling, in SOX2-expressing precursors resulted in precocious neuronal differentiation alongside the depletion of the precursor pool (Ehm et al., 2010). Similarly, RE1 silencing transcription factor (REST) deficiency in RGL cells leads to a transient increase in neurogenesis and a decrease in RGL cells (Gao et al., 2011). Loss of *Pax6* results in a smaller GFAP$^+$ RGL cell pool, with aberrant radial processes, coupled with abnormal neuronal progenitors, indicating impaired neurogenesis (Maekawa et al., 2005). Deletion of COUP-TFI (*Nr2f1*) caused an increase in astrogliogenesis at the expense of neurogenesis, but no loss of RGL cells was observed (Bonzano et al., 2018). Because of the link between ZEB1 and SOX2 in glioblastoma (Singh et al., 2017), it is worth comparing the functions of both proteins in adult neurogenesis. Interestingly, loss of *Sox2* decreased the RGL cell pool and cell proliferation but had only limited effects on neurogenesis (Favaro et al., 2009). This may be because of a cell context-dependent function with SOX2

inhibiting the expression of NeuroD1 in RGL cells while inducing NeuroD1 when expressed in IPCs (Kuwabara et al., 2009). Here, we show that *Zeb1* loss causes differentiation of RGL cells into the neuronal lineage, thus inducing a transient increase in neurogenesis. The increase in neuroblast production is further amplified by decreased neuronal apoptosis during maturation; thus, elevated neuron numbers are a result of both production and survival.

RGL cells also generate astrocytes (Bonaguidi et al., 2011; Bonzano et al., 2018; Gebara et al., 2016; Steiner et al., 2004). If lineage commitment of differentiating *Zeb1*$^{-/-}$ RGL cells is stochastic, numbers of both newborn neurons and astrocytes should increase. Contrastingly, we found that astrocyte numbers decreased in the SGZ, but not in the remaining DG, and that apoptosis of astrocytes was not different between control and *Zeb1*$^{-/-}$ mice. This indicates that the differentiation of *Zeb1*$^{-/-}$ RGL cells is not random. Clonal analysis showed that the remaining active clones in *Zeb1*$^{-/-}$ mice specifically lacked astrocytes, confirming increased neurogenesis at the expense of astrogliogenesis. Whether ZEB1 suppresses neurogenesis or has direct functions in astrocyte lineage commitment remains to be elucidated. We further show that *Zeb1* deletion correlates with increased symmetric cell divisions of RGL cells *in vitro* and *in vivo*. Notably, ZEB1 is asymmetrically distributed during cell division in precancerous adenomas (Liu et al., 2018). Increased symmetric divisions following *Zeb1* loss are compatible with premature depletion of RGL cells coupled with the production of lineage-committed progenitors. This could indicate that symmetric division of RGL cells favors neuronal differentiation, while astrocyte progenies are generated through asymmetric division, but further research is needed to validate this.

Lower astrocyte counts in *Zeb1*$^{-/-}$ mice correlated with increased neurogenesis, changes in RGL cell division type, and decreased expression of the ets domain transcription factor ETV5. We identify ETV5 as a direct transcriptional target of ZEB1 that is predominantly expressed in RGL cells within the SGZ. Importantly, ETV5 is a key regulator of astroglial fate and asymmetrical stem cell divisions (Ahmad et al., 2019; Breunig et al., 2015; Li et al., 2012). Aberrant expression of ETV5 during neurodevelopment results in increased gliogenesis (Newton et al., 2018), and targeted expression of ETV5 blocked neural stem cell depletion and tumorigenesis in a mouse model of glioma formation (Breunig et al., 2015). In our model, targeted expression of ETV5 in *Zeb1*$^{-/-}$ clones *in vitro* increased asymmetrical divisions, decreased cell division times, and reduced the number of neuronal progenies. Therefore, ETV5 can rescue the effects of *Zeb1* loss on cell division type and neuronal differentiation.

In conclusion, ZEB1 demarcates active from quiescent RGL cells, regulates asymmetrical cell division and thus self-renewal of RGL cells, and directly activates expression of the lineage transcription factor ETV5.

STAR★METHODS

Detailed methods are provided in the online version of this paper and include the following:

- KEY RESOURCES TABLE
- RESOURCE AVAILABILITY
  - Lead contact
  - Materials availability
  - Data and code availability
- EXPERIMENTAL MODEL AND SUBJECT DETAILS
  - Ethics statement
  - Animal husbandry
  - Animal lines
- METHOD DETAILS
  - Mouse transgenic lines and genotyping
  - Tamoxifen administration
  - 5-ethynyl-2′deoxyuridine (EdU) treatment
  - Tissue processing, immunostaining, and confocal imaging
  - Clonal analysis
  - Cleavage plane measurements
  - Brain slice preparation, electrophysiology and 2-photon imaging
  - *In vitro* primary cultures and molecular biology
  - *In vitro* time-lapse imaging of primary hippocampal cells
- QUANTIFICATION AND STATISTICAL ANALYSIS

SUPPLEMENTAL INFORMATION

Supplemental information can be found online at https://doi.org/10.1016/j.celrep.2021.109588.

ACKNOWLEDGMENTS

Funding was provided by the Medical Research Council (MR/S007709/1 to F.A.S.), the German Research Foundation (BR4145/1-1 and 4145/2-1 to S.B.; SFB850, A4 and B2 to M.P.S.; FOR 2438/project 4, BR1399/9-1, 1399/10-1, and 1399/13-1 to T.B.), the Wilhelm-Sander Foundation (2020.039.1 to S.B.); the EU (Horizon 2020, grant 861196, PRECODE to M.P.S.), and Cancer Research UK (PhD studentship to B.G.). We thank D. Siebzehnruebl for technical assistance. Illustrations (Figure 7A, graphical abstract) were generated by Art & Science - Dorotea Fracchiolla.

AUTHOR CONTRIBUTIONS

Conceptualization, F.A.S.; methodology, B.G. and F.A.S.; investigation, A.C.E., A.J.-P., B.G., D.P., and V.E.; resources, M.P.S., S.B., and T.B.; writing – original draft, B.G. and F.A.S.; writing – review and editing, B.G., D.P., and F.A.S.; supervision, F.A.S.; funding acquisition, F.A.S., M.P.S., S.B., and T.B.

DECLARATION OF INTERESTS

The authors declare no competing interests.

Received: November 6, 2020
Revised: May 27, 2021
Accepted: July 30, 2021
Published: August 24, 2021

REFERENCES

Acloque, H., Adams, M.S., Fishwick, K., Bronner-Fraser, M., and Nieto, M.A. (2009). Epithelial-mesenchymal transitions: the importance of changing cell state in development and disease. J. Clin. Invest. *119*, 1438–1449.

Ahmad, S.T., Rogers, A.D., Chen, M.J., Dixit, R., Adnani, L., Frankiw, L.S., Lawn, S.O., Blough, M.D., Alshehri, M., Wu, W., et al. (2019). Capicua regulates

CellPress
OPEN ACCESS

neural stem cell proliferation and lineage specification through control of Ets factors. Nat. Commun. *10*, 2000.

Bani-Yaghoub, M., Tremblay, R.G., Lei, J.X., Zhang, D., Zurakowski, B., Sandhu, J.K., Smith, B., Ribecco-Lutkiewicz, M., Kennedy, J., Walker, P.R., and Sikorska, M. (2006). Role of Sox2 in the development of the mouse neocortex. Dev. Biol. *295*, 52–66.

Bonaguidi, M.A., Wheeler, M.A., Shapiro, J.S., Stadel, R.P., Sun, G.J., Ming, G.L., and Song, H. (2011). In vivo clonal analysis reveals self-renewing and multipotent adult neural stem cell characteristics. Cell *145*, 1142–1155.

Bonzano, S., Crisci, I., Podlesny-Drabiniok, A., Rolando, C., Krezel, W., Studer, M., and De Marchis, S. (2018). Neuron-Astroglia Cell Fate Decision in the Adult Mouse Hippocampal Neurogenic Niche Is Cell-Intrinsically Controlled by COUP-TFI In Vivo. Cell Rep. *24*, 329–341.

Brabletz, S., Lasierra Losada, M., Schmalhofer, O., Mitschke, J., Krebs, A., Brabletz, T., and Stemmler, M.P. (2017). Generation and characterization of mice for conditional inactivation of Zeb1. Genesis *55*, e23024.

Breunig, J.J., Levy, R., Antonuk, C.D., Molina, J., Dutra-Clarke, M., Park, H., Akhtar, A.A., Kim, G.B., Hu, X., Bannykh, S.I., et al. (2015). Ets Factors Regulate Neural Stem Cell Depletion and Gliogenesis in Ras Pathway Glioma. Cell Rep. *12*, 258–271.

Brown, J.P., Couillard-Després, S., Cooper-Kuhn, C.M., Winkler, J., Aigner, L., and Kuhn, H.G. (2003). Transient expression of doublecortin during adult neurogenesis. J. Comp. Neurol. *467*, 1–10.

Chai, H., Diaz-Castro, B., Shigetomi, E., Monte, E., Octeau, J.C., Yu, X., Cohn, W., Rajendran, P.S., Vondriska, T.M., Whitelegge, J.P., et al. (2017). Neural Circuit-Specialized Astrocytes: Transcriptomic, Proteomic, Morphological, and Functional Evidence. Neuron *95*, 531–549.e9.

Chotteau-Lelievre, A., Montesano, R., Soriano, J., Soulie, P., Desbiens, X., and de Launoit, Y. (2003). PEA3 transcription factors are expressed in tissues undergoing branching morphogenesis and promote formation of duct-like structures by mammary epithelial cells in vitro. Dev. Biol. *259*, 241–257.

Coras, R., Siebzehnrubl, F.A., Pauli, E., Huttner, H.B., Njunting, M., Kobow, K., Villmann, C., Hahnen, E., Neuhuber, W., Weigel, D., et al. (2010). Low proliferation and differentiation capacities of adult hippocampal stem cells correlate with memory dysfunction in humans. Brain *133*, 3359–3372.

Dayer, A.G., Ford, A.A., Cleaver, K.M., Yassaee, M., and Cameron, H.A. (2003). Short-term and long-term survival of new neurons in the rat dentate gyrus. J. Comp. Neurol. *460*, 563–572.

Edwards, L.A., Woolard, K., Son, M.J., Li, A., Lee, J., Ene, C., Mantey, S.A., Maric, D., Song, H., Belova, G., et al. (2011). Effect of brain- and tumor-derived connective tissue growth factor on glioma invasion. J. Natl. Cancer Inst. *103*, 1162–1178.

Ehm, O., Göritz, C., Covic, M., Schäffner, I., Schwarz, T.J., Karaca, E., Kempkes, B., Kremmer, E., Pfrieger, F.W., Espinosa, L., et al. (2010). RBPJkappa-dependent signaling is essential for long-term maintenance of neural stem cells in the adult hippocampus. J. Neurosci. *30*, 13794–13807.

Encinas, J.M., Michurina, T.V., Peunova, N., Park, J.H., Tordo, J., Peterson, D.A., Fishell, G., Koulakov, A., and Enikolopov, G. (2011). Division-coupled astrocytic differentiation and age-related depletion of neural stem cells in the adult hippocampus. Cell Stem Cell *8*, 566–579.

Falk, S., Bugeon, S., Ninkovic, J., Pilz, G.A., Postiglione, M.P., Cremer, H., Knoblich, J.A., and Götz, M. (2017). Time-Specific Effects of Spindle Positioning on Embryonic Progenitor Pool Composition and Adult Neural Stem Cell Seeding. Neuron *93*, 777–791.e3.

Favaro, R., Valotta, M., Ferri, A.L., Latorre, E., Mariani, J., Giachino, C., Lancini, C., Tosetti, V., Ottolenghi, S., Taylor, V., and Nicolis, S.K. (2009). Hippocampal development and neural stem cell maintenance require Sox2-dependent regulation of Shh. Nat. Neurosci. *12*, 1248–1256.

Fornes, O., Castro-Mondragon, J.A., Khan, A., van der Lee, R., Zhang, X., Richmond, P.A., Modi, B.P., Correard, S., Gheorghe, M., Baranašić, D., et al. (2020). JASPAR 2020: update of the open-access database of transcription factor binding profiles. Nucleic Acids Res. *48* (*D1*), D87–D92.

Gage, F.H. (2019). Adult neurogenesis in mammals. Science *364*, 827–828.

Gao, Z., Ure, K., Ding, P., Nashaat, M., Yuan, L., Ma, J., Hammer, R.E., and Hsieh, J. (2011). The master negative regulator REST/NRSF controls adult neurogenesis by restraining the neurogenic program in quiescent stem cells. J. Neurosci. *31*, 9772–9786.

Gebara, E., Bonaguidi, M.A., Beckervordersandforth, R., Sultan, S., Udry, F., Gijs, P.J., Lie, D.C., Ming, G.L., Song, H., and Toni, N. (2016). Heterogeneity of Radial Glia-Like Cells in the Adult Hippocampus. Stem Cells *34*, 997–1010.

Goossens, S., Vandamme, N., Van Vlierberghe, P., and Berx, G. (2017). EMT transcription factors in cancer development re-evaluated: Beyond EMT and MET. Biochim. Biophys. Acta Rev. Cancer *1868*, 584–591.

Harris, L., Rigo, P., Stiehl, T., Gaber, Z.B., Austin, S.H.L., Masdeu, M.D.M., Edwards, A., Urbán, N., Marciniak-Czochra, A., and Guillemot, F. (2021). Coordinated changes in cellular behavior ensure the lifelong maintenance of the hippocampal stem cell population. Cell Stem Cell *28*, 863–876.e6.

Hilsenbeck, O., Schwarzfischer, M., Skylaki, S., Schauberger, B., Hoppe, P.S., Loeffler, D., Kokkaliaris, K.D., Hastreiter, S., Skylaki, E., Filipczyk, A., et al. (2016). Software tools for single-cell tracking and quantification of cellular and molecular properties. Nat. Biotechnol. *34*, 703–706.

Hoang-Minh, L.B., Siebzehnrubl, F.A., Yang, C., Suzuki-Hatano, S., Dajac, K., Loche, T., Andrews, N., Schmoll Massari, M., Patel, J., Amin, K., et al. (2018). Infiltrative and drug-resistant slow-cycling cells support metabolic heterogeneity in glioblastoma. EMBO J. *37*, e98772.

Hochgerner, H., Zeisel, A., Lönnerberg, P., and Linnarsson, S. (2018). Conserved properties of dentate gyrus neurogenesis across postnatal development revealed by single-cell RNA sequencing. Nat. Neurosci. *21*, 290–299.

Jahn, H.M., Kasakow, C.V., Helfer, A., Michely, J., Verkhratsky, A., Maurer, H.H., Scheller, A., and Kirchhoff, F. (2018). Refined protocols of tamoxifen injection for inducible DNA recombination in mouse astroglia. Sci. Rep. *8*, 5913.

Jimenez-Pascual, A., Hale, J.S., Kordowski, A., Pugh, J., Silver, D.J., Bayik, D., Roversi, G., Alban, T.J., Rao, S., Chen, R., et al. (2019). ADAMDEC1 Maintains a Growth Factor Signaling Loop in Cancer Stem Cells. Cancer Discov. *9*, 1574–1589.

Kempermann, G., Jessberger, S., Steiner, B., and Kronenberg, G. (2004). Milestones of neuronal development in the adult hippocampus. Trends Neurosci. *27*, 447–452.

Kempermann, G., Gage, F.H., Aigner, L., Song, H., Curtis, M.A., Thuret, S., Kuhn, H.G., Jessberger, S., Frankland, P.W., Cameron, H.A., et al. (2018). Human Adult Neurogenesis: Evidence and Remaining Questions. Cell Stem Cell *23*, 25–30.

Kuwabara, T., Hsieh, J., Muotri, A., Yeo, G., Warashina, M., Lie, D.C., Moore, L., Nakashima, K., Asashima, M., and Gage, F.H. (2009). Wnt-mediated activation of NeuroD1 and retro-elements during adult neurogenesis. Nat. Neurosci. *12*, 1097–1105.

Lazutkin, A., Podgorny, O., and Enikolopov, G. (2019). Modes of division and differentiation of neural stem cells. Behav. Brain Res. *374*, 112118.

Li, X., Newbern, J.M., Wu, Y., Morgan-Smith, M., Zhong, J., Charron, J., and Snider, W.D. (2012). MEK Is a Key Regulator of Gliogenesis in the Developing Brain. Neuron *75*, 1035–1050.

Ligon, K.L., Huillard, E., Mehta, S., Kesari, S., Liu, H., Alberta, J.A., Bachoo, R.M., Kane, M., Louis, D.N., Depinho, R.A., et al. (2007). Olig2-regulated lineage-restricted pathway controls replication competence in neural stem cells and malignant glioma. Neuron *53*, 503–517.

Liu, Y., Siles, L., Lu, X., Dean, K.C., Cuatrecasas, M., Postigo, A., and Dean, D.C. (2018). Mitotic polarization of transcription factors during asymmetric division establishes fate of forming cancer cells. Nat. Commun. *9*, 2424.

Liu, J., Liu, Y., Shao, J., Li, Y., Qin, L., Shen, H., Xie, Y., Xia, W., and Gao, W.Q. (2019). Zeb1 is important for proper cleavage plane orientation of dividing progenitors and neuronal migration in the mouse neocortex. Cell Death Differ. *26*, 2479–2492.

Madisen, L., Zwingman, T.A., Sunkin, S.M., Oh, S.W., Zariwala, H.A., Gu, H., Ng, L.L., Palmiter, R.D., Hawrylycz, M.J., Jones, A.R., et al. (2010). A robust and high-throughput Cre reporting and characterization system for the whole mouse brain. Nat. Neurosci. *13*, 133–140.

Maekawa, M., Takashima, N., Arai, Y., Nomura, T., Inokuchi, K., Yuasa, S., and Osumi, N. (2005). Pax6 is required for production and maintenance of progenitor cells in postnatal hippocampal neurogenesis. Genes Cells *10*, 1001–1014.

Martín-Suárez, S., Valero, J., Muro-García, T., and Encinas, J.M. (2019). Phenotypical and functional heterogeneity of neural stem cells in the aged hippocampus. Aging Cell *18*, e12958.

Ming, G.L., and Song, H. (2011). Adult neurogenesis in the mammalian brain: significant answers and significant questions. Neuron *70*, 687–702.

Mori, T., Tanaka, K., Buffo, A., Wurst, W., Kühn, R., and Götz, M. (2006). Inducible gene deletion in astroglia and radial glia–a valuable tool for functional and lineage analysis. Glia *54*, 21–34.

Newton, K., Dugger, D.L., Sengupta-Ghosh, A., Ferrando, R.E., Chu, F., Tao, J., Lam, W., Haller, S., Chan, S., Sa, S., et al. (2018). Ubiquitin ligase COP1 coordinates transcriptional programs that control cell type specification in the developing mouse brain. Proc. Natl. Acad. Sci. USA *115*, 11244–11249.

Ohayon, D., Garcès, A., Joly, W., Soukkarieh, C., Takagi, T., Sabourin, J.C., Agius, E., Darling, D.S., De Santa Barbara, P., Higashi, Y., et al. (2016). Onset of Spinal Cord Astrocyte Precursor Emigration from the Ventricular Zone Involves the Zeb1 Transcription Factor. Cell Rep. *17*, 1473–1481.

Ortega, F., Berninger, B., and Costa, M.R. (2013). Primary culture and live imaging of adult neural stem cells and their progeny. Methods Mol. Biol. *1052*, 1–11.

Palmer, T.D., Takahashi, J., and Gage, F.H. (1997). The adult rat hippocampus contains primordial neural stem cells. Mol. Cell. Neurosci. *8*, 389–404.

Peinado, H., Olmeda, D., and Cano, A. (2007). Snail, Zeb and bHLH factors in tumour progression: an alliance against the epithelial phenotype? Nat. Rev. Cancer *7*, 415–428.

Petrik, D., Myoga, M.H., Grade, S., Gerkau, N.J., Pusch, M., Rose, C.R., Grothe, B., and Götz, M. (2018). Epithelial Sodium Channel Regulates Adult Neural Stem Cell Proliferation in a Flow-Dependent Manner. Cell Stem Cell *22*, 865–878.e8.

Pilz, G.A., Bottes, S., Betizeau, M., Jörg, D.J., Carta, S., Simons, B.D., Helmchen, F., and Jessberger, S. (2018). Live imaging of neurogenesis in the adult mouse hippocampus. Science *359*, 658–662.

Rosmaninho, P., Mükusch, S., Piscopo, V., Teixeira, V., Raposo, A.A., Warta, R., Bennewitz, R., Tang, Y., Herold-Mende, C., Stifani, S., et al. (2018). Zeb1 potentiates genome-wide gene transcription with Lef1 to promote glioblastoma cell invasion. EMBO J. *37*, e97115.

Ryu, J.R., Hong, C.J., Kim, J.Y., Kim, E.K., Sun, W., and Yu, S.W. (2016). Control of adult neurogenesis by programmed cell death in the mammalian brain. Mol. Brain *9*, 43.

Seri, B., García-Verdugo, J.M., McEwen, B.S., and Alvarez-Buylla, A. (2001). Astrocytes give rise to new neurons in the adult mammalian hippocampus. J. Neurosci. *21*, 7153–7160.

Siebzehnrubl, F.A., Silver, D.J., Tugertimur, B., Deleyrolle, L.P., Siebzehnrubl, D., Sarkisian, M.R., Devers, K.G., Yachnis, A.T., Kupper, M.D., Neal, D., et al. (2013). The ZEB1 pathway links glioblastoma initiation, invasion and chemoresistance. EMBO Mol. Med. *5*, 1196–1212.

Siebzehnrübl, F.A., Raber, K.A., Urbach, Y.K., Schulze-Krebs, A., Canneva, F., Moceri, S., Habermeyer, J., Achoui, D., Gupta, B., Steindler, D.A., et al. (2018). Early postnatal behavioral, cellular, and molecular changes in models of Huntington disease are reversible by HDAC inhibition. Proc. Natl. Acad. Sci. USA *115*, E8765–E8774.

Sierra, A., Encinas, J.M., Deudero, J.J., Chancey, J.H., Enikolopov, G., Overstreet-Wadiche, L.S., Tsirka, S.E., and Maletic-Savatic, M. (2010). Microglia shape adult hippocampal neurogenesis through apoptosis-coupled phagocytosis. Cell Stem Cell *7*, 483–495.

Singh, S., Howell, D., Trivedi, N., Kessler, K., Ong, T., Rosmaninho, P., Raposo, A.A., Robinson, G., Roussel, M.F., Castro, D.S., and Solecki, D.J. (2016). Zeb1 controls neuron differentiation and germinal zone exit by a mesenchymal-epithelial-like transition. eLife *5*, e12717.

Singh, D.K., Kollipara, R.K., Vemireddy, V., Yang, X.L., Sun, Y., Regmi, N., Klingler, S., Hatanpaa, K.J., Raisanen, J., Cho, S.K., et al. (2017). Oncogenes Activate an Autonomous Transcriptional Regulatory Circuit That Drives Glioblastoma. Cell Rep. *18*, 961–976.

Spaderna, S., Schmalhofer, O., Wahlbuhl, M., Dimmler, A., Bauer, K., Sultan, A., Hlubek, F., Jung, A., Strand, D., Eger, A., et al. (2008). The transcriptional repressor ZEB1 promotes metastasis and loss of cell polarity in cancer. Cancer Res. *68*, 537–544.

Steiner, B., Kronenberg, G., Jessberger, S., Brandt, M.D., Reuter, K., and Kempermann, G. (2004). Differential regulation of gliogenesis in the context of adult hippocampal neurogenesis in mice. Glia *46*, 41–52.

Stemmler, M.P., Eccles, R.L., Brabletz, S., and Brabletz, T. (2019). Non-redundant functions of EMT transcription factors. Nat. Cell Biol. *21*, 102–112.

Suh, H., Consiglio, A., Ray, J., Sawai, T., D'Amour, K.A., and Gage, F.H. (2007). In vivo fate analysis reveals the multipotent and self-renewal capacities of Sox2+ neural stem cells in the adult hippocampus. Cell Stem Cell *1*, 515–528.

Takagi, T., Moribe, H., Kondoh, H., and Higashi, Y. (1998). DeltaEF1, a zinc finger and homeodomain transcription factor, is required for skeleton patterning in multiple lineages. Development *125*, 21–31.

Trent, S., Hall, J., Connelly, W.M., and Errington, A.C. (2019). *Cyfip1* Haploinsufficiency Does Not Alter $GABA_A$ Receptor δ-Subunit Expression and Tonic Inhibition in Dentate Gyrus PV^+ Interneurons and Granule Cells. eNeuro *6*, ENEURO.0364-18.2019.

Wang, B., Krall, E.B., Aguirre, A.J., Kim, M., Widlund, H.R., Doshi, M.B., Sicinska, E., Sulahian, R., Goodale, A., Cowley, G.S., et al. (2017). ATXN1L, CIC, and ETS Transcription Factors Modulate Sensitivity to MAPK Pathway Inhibition. Cell Rep. *18*, 1543–1557.

Wang, H., Xiao, Z., Zheng, J., Wu, J., Hu, X.L., Yang, X., and Shen, Q. (2019). ZEB1 Represses Neural Differentiation and Cooperates with CTBP2 to Dynamically Regulate Cell Migration during Neocortex Development. Cell Rep. *27*, 2335–2353.e6.

White, C.W., 3rd, Fan, X., Maynard, J.C., Wheatley, E.G., Bieri, G., Couthouis, J., Burlingame, A.L., and Villeda, S.A. (2020). Age-related loss of neural stem cell O-GlcNAc promotes a glial fate switch through STAT3 activation. Proc. Natl. Acad. Sci. USA *117*, 22214–22224.

Zeisel, A., Muñoz-Manchado, A.B., Codeluppi, S., Lönnerberg, P., La Manno, G., Juréus, A., Marques, S., Munguba, H., He, L., Betsholtz, C., et al. (2015). Brain structure. Cell types in the mouse cortex and hippocampus revealed by single-cell RNA-seq. Science *347*, 1138–1142.

STAR★METHODS

KEY RESOURCES TABLE

REAGENT or RESOURCE	SOURCE	IDENTIFIER
Antibodies		
Rabbit anti-ARHGEF2	Invitrogen	Cat#720323: RRID: AB_2633257
Rabbit anti-Cleaved caspase 3	Cell Signaling	Cat#9664S; RRID: AB_2070042
Chicken anti-Doublecortin (DCX)	Aves Lab	Cat#DCX; RRID: AB_2313540
Guinea pig anti-DCX	Millipore	Cat#AB2253; RRID: AB_1586992
Rabbit anti-ETV5	Invitrogen	Cat#PA5-30023; RRID: AB_2313540
Chicken anti-GFAP	Encor Biotechnology	Cat#CPCA-GFAP; RRID: AB_2109953
Mouse anti-GFAP	Sigma-Aldrich	Cat#G6171; RRID: AB_1840893
Rabbit anti-GFAP	Dako	Cat#Z0334; RRID: AB_10013382
Rabbit anti-INSC	Proteintech	Cat# 20973-1-AP; RRID: AB_10951111
Chicken anti-Ki67	Encor	Cat#CPCA-Ki67; RRID: AB_2637049
Rabbit anti-MCM2	Abcam	Cat#ab4461; RRID: AB_304470
Mouse anti-NeuN	Merck	Cat# MAB377; RRID: AB_2298772
Rabbit anti-RAB10	Cell Signaling	Cat#8127T; RRID: AB_10828219
Rabbit anti-S100β	NeoMarkers	Cat#AB-044-AO; RRID: AB_60518
Rabbit anti-TBR2	Abcam	Cat#ab23345; RRID: AB_778267
Rabbit anti-ZEB1	Sigma-Aldrich	Cat#HPA027524; RRID: AB_1844977
Goat anti-chicken Alexa Fluor 488	Invitrogen	Cat#A11039; RRID: AB_142924
Donkey anti-mouse Alexa Fluor 488	Invitrogen	Cat#A21202; RRID: AB_141607
Goat anti-mouse Alexa Fluor 405	Invitrogen	Cat#A31553; RRID: AB_221604
Donkey anti-rabbit Alexa Fluor 488	Invitrogen	Cat#A21206; RRID: AB_2535792
Donkey anti-rabbit Alexa Fluor 647	Invitrogen	Cat#A32795; RRID: AB_2762835
Donkey anti-rabbit Alexa Fluor 594	Invitrogen	Cat# A32754; RRID: AB_2762827
Donkey anti-rabbit IgG Fab fragments	Jackson ImmunoResearch	Cat# 711-007-003; RRID:AB_2340587
Bacterial and virus strains		
Lentiviral plasmid for ETV5 expression (pLX_TRC311 ETV5)	Wang et al., 2017	Addgene plasmid # 74984; http://addgene.org/74984/ ; RRID:Addgene_74984
Chemical, peptides, and recombinant proteins		
Optimal Cutting Temperature (OCT) Compound	Thermo Fisher Scientific	Cat#6502
Fish Skin Gelatin	Sigma Aldrich	Cat#G7765
Triton X-100	Thermo Fisher Scientific	Cat#13454259
10XPBS	Thermo Fisher Scientific	Cat#70011044
Hoechst-33342	Thermo Fisher Scientific	Cat#62249
Prolong Diamond Antifade Mountant	Thermo Fisher Scientific	Cat#P36961
DMEM/F12 with Glutamax	Thermo Fisher Scientific	Cat#11320033
recombinant human Insulin	Sigma-Aldrich	Cat#I5500
recombinant human Transferrin	Sigma-Aldrich	Cat#T8158
Putrescine	Sigma-Aldrich	Cat#P5780
Sodium Selenite	Sigma-Aldrich	Cat#S5261
Progesterone	Sigma-Aldrich	Cat#P8783
Bovine Serum Albumin (BSA) fraction V	Thermo Fisher Scientific	Cat#12737119
B27 supplement	Thermo Fisher Scientific	Cat#17504044
Penicillin-Streptomycin solution	Thermo Fisher Scientific	Cat#15140122

(*Continued on next page*)

Continued

REAGENT or RESOURCE	SOURCE	IDENTIFIER
HBSS	Thermo Fisher Scientific	Cat#24020117
Papain	Roche	Cat#10108014001
Dispase II	Roche	Cat#165859
DNase I	Roche	Cat#10104159001
Hyaluronidase	Sigma-Aldrich	Cat#H3884
Trypsin	Sigma-Aldrich	Cat#T9201
Heparin	Sigma-Aldrich	Cat#H4784
recombinant human EGF	Peprotech	Cat#AF-100-15
recombinant human FGF-2	Peprotech	Cat#AF-100-18C
Poly-D-lysine	Sigma-Aldrich	Cat#P6407
Laminin	Sigma-Aldrich	Cat#L2020
Accumax	Thermo Fisher Scientific	Cat#00-4666-56
Tamoxifen	Sigma-Aldrich	Cat#T5648
4-Hydroxy-Tamoxifen	Sigma-Aldrich	Cat#SML1666
Corn oil	Sigma-Aldrich	Cat#C8267
Formaldehyde	Santa Cruz Biotechnology	Cat#sc-203049
Critical commercial assays		
EdU Click 488 kit	Sigma-Aldrich	Cat#BCK-EDU488
SimpleChIP kit	Cell Signaling Technologies	Cat#91820
MyTaq Extract-PCR kit	Bioline	Cat#BIO-21127
MyTaq HS Red Mix 2X	Bioline	Cat#BIO-25047
Experimental models: Organisms/strains		
Mouse: GLAST::CreER^{T2}	M. Götz, Munich	N/A
Mouse: Rosa26$^{lox-stop-lox-tdTomato}$ (Ai9)	O. Sansom, Glasgow	N/A
Mouse: Zeb1$^{flox/flox}$	T. Brabletz, Erlangen	N/A
Oligonucleotides		
See Table S3 for oligonucleotide information.		
Deposited data		
Single-cell RNA-Seq data from RGL cells and astrocytes	Chai et al., 2017	GEO: GSE94010
Single-cell RNA-Seq data from dentate gyrus	Hochgerner et al., 2018	GEO: GSE95315
Chip-Seq data of glioblastoma cells	Rosmaninho et al., 2018	EMBL accession: E-MTAB-5541
Single-cell RNA-Seq data from cortex and hippocampus	Zeisel et al., 2015	GEO: GSE60361
Software and algorithms		
ZEN microscopy software	Zeiss	https://www.zeiss.com/microscopy/int/products/microscope-software/zen.html
ImageJ v2.52K	ImageJ NIH	https://imagej.net/
Neurolucida 360/Explorer	MBF Bioscience	N/A
GraphPad Prism v9.1	GraphPad Software, Inc.	https://www.graphpad.com/
The Tracing Tool (tTt) software v3.4.4	Hilsenbeck et al., 2016	https://bsse.ethz.ch/csd/software/ttt-and-qtfy.html

RESOURCE AVAILABILITY

Lead contact

Further information and requests for resources and regents should be directed to and will be fulfilled by the lead contact, Florian Siebzehnrubl (fas@cardiff.ac.uk).

Materials availability

All materials and lines generated in this study are available from the lead contact.

Data and code availability

- This paper analyzes existing, publicly available data. The accession numbers for the datasets are listed in the Key resources table.
- This paper does not report original code.
- Any additional information required to reanalyze the data reported in this paper is available from the lead contact upon request.

EXPERIMENTAL MODEL AND SUBJECT DETAILS

Ethics statement

All mouse husbandry and experiments were carried out in accordance with UK Home Office regulations and the Animals (Scientific Procedures) Act 1986.

Animal husbandry

All mice were group-housed in 12-hour light/dark cycles in filter top cages and given free access to food (Teklad 2919 irradiated 19% protein extruded diet, Envigo) and water. Cages were cleaned weekly, and nesting material as well as plastic tunnels were provided for environmental enrichment.

Animal lines

All mice were maintained on a mixed genetic background. Both male and female mice were used for all experiments and randomly allocated to experimental groups.

The GLAST::CreER^{T2}, Rosa26$^{lox\text{-}stop\text{-}lox\text{-}tdTomato}$ (Ai9), and Zeb1$^{flox/flox}$ transgenic mouse lines have been previously described (Brabletz et al., 2017; Madisen et al., 2010; Mori et al., 2006). The GLAST::CreER^{T2}, Rosa26$^{lox\text{-}stop\text{-}lox\text{-}tdTomato}$ and GLAST::CreER^{T2}, Rosa26$^{lox\text{-}stop\text{-}lox\text{-}tdTomato}$, Zeb1$^{flox/flox}$ mouse lines were derived in this study, as described below.

METHOD DETAILS

Mouse transgenic lines and genotyping

A transgenic mouse line with loxP sites flanking exon 6 of the *Zeb1* gene (Brabletz et al., 2017), was crossed with the GLAST::CreER^{T2} mouse line (kind gift from M. Götz, Munich; (Mori et al., 2006)) and further crossbred with the Rosa26$^{lox\text{-}stop\text{-}lox\text{-}tdTomato}$ strain (kind gift from O. Sansom, Glasgow; (Madisen et al., 2010)). GLAST::CreER^{T2}-Rosa26$^{lox\text{-}stop\text{-}lox\text{-}tdTomato}$ mice with wild-type ZEB1 expression were used as controls (referred to as the control strain).

For genotyping, ear biopsies were taken at the time of weaning and genomic DNA was extracted using the MyTaq Extract-PCR kit, according to the manufacturer's protocol. Subsequently, the genomic DNA was analyzed by PCR using the MyTaq HS Red Mix 2X, according to the manufacturer's protocol. Primers used for Glast::Cre PCR were: Glast-F 5′-GAGGACTTGGCTAGGCTCTGAG-3′, Glast-R 5′- GAGGAGATCCTGACCGATCAGTT-3′, and Cre-R 5′-GGTGTACGGTCAGTAAATTGGAC-3′ (WT 700bp, mt 400bp). Primers used for Zeb1 flox PCR were: Zeb1 fl-F 5′-CGTGATGGAGCCAGAATCTGACCC-3′, Zeb1 fl-R 5′-GCCCTGTCTTTCTCAG-CAGTGTGG-3′, Zeb1 exon 6 deleted-R 5′-GCCATCTCACCAGCCCTTACTGTGC-3′ (WT 230bp, floxed 295bp, exon 6 deletion 367 bp). Primers used for tdTomato PCR were: WT-F 5′-AAGGGAGCTGCAGTGGAGTA-3′, WT-R 5′-GGCATTAAAGCAGCG-TATCC-3′, mt-F 5′-CCGAAAATCTGTGGGAAGTC-3′, mt-R 5′-CTGTTCCTGTACGGCATGG-3′ (WT 297bp, mt 196bp).

Tamoxifen administration

A stock solution (20 mg/mL) of tamoxifen was prepared by dissolving the compound in corn oil at 70°C in a ThermoMixer (Eppendorf) for 30-60 mins, which was subsequently aliquoted to be stored at −20°C to avoid freeze-thaw cycles. For regular transgene induction, a 2 mg dose of tamoxifen was injected intraperitoneally into 4-5 week-old mice daily for five consecutive days (Jahn et al., 2018). For clonal analysis, a 50 μg dose of tamoxifen in corn oil was injected i.p. into 4-5 week-old mice daily for three consecutive days. Mice were transcardially perfused with 2% formaldehyde in PBS and the brains were harvested for histological analysis at time points indicated in the text.

5-ethynyl-2′deoxyuridine (EdU) treatment

Mice were injected i.p. with five 2 mg doses of tamoxifen over three consecutive days (Jahn et al., 2018), followed by five i.p. injections with 50 mg/kg EdU over three consecutive days. Two weeks following tamoxifen administration, mice were transcardially perfused and the brains were harvested for histological analysis and processed for EdU detection using the EdU Click 488 kit according to the manufacturer's protocol.

Tissue processing, immunostaining, and confocal imaging

Tissue was processed as previously described (Jimenez-Pascual et al., 2019). Briefly, following harvesting, an overnight post-fixation in 2% formaldehyde, and an additional overnight wash in 1XPBS, the tissue was dehydrated in 30% [w/v] sucrose solution. The tissue was then embedded in Optimal Cutting Temperature compound (OCT) and frozen for cryosectioning. Subseqently, 30 μm thick coronal sections were cut on a Leica CM1860UV cryostat (Leica Biosystems) and maintained in serial order, with one section per well in a 96-well plate.

For immunofluorescence staining, chosen sections were transferred to a 24-well plate containing 500 μL of 1XPSB with 0.1% Triton X-100 [v/v] (PBS-T) per well, and washed for 10 mins on a Rotamax 120 (Heidolph Instruments; all incubations and wash steps hereafter were carried out on the rotating platform) with 20 rotations/min at RT. Subsequently, the PBS-T was removed using a fine Pasteur pipette and 500 μL of Fish-skin gelatin buffer (0.2 [v/v] fish-skin gelatin, 1% [w/v] BSA, and 0.02% [w/v] sodium azide in 1XPBS) with 0.1% Triton X-100 [v/v] (FSB-T) was added for tissue permeabilization and blocking, with incubation at RT for 1 hour. Meanwhile, primary antibodies were prepared by appropriate dilution (cleaved caspase 3 1:250; DCX 1:250; ETV5 1:500; chicken anti-GFAP 1:1000; mouse anti-GFAP 1:250; Ki67 1:500; MCM2 1:500; NeuN 1:500; S100β 1:250; TBR2 1:500; ZEB1 1:500) in 250 μL FSB-T in 1.5 mL reaction tubes; the FSB-T was removed and the diluted antibodies were pipetted into the well with the tissue and incubated overnight at 4°C. The following morning, 5X 5-minute washes were carried out in PBS-T, after which fluorophore-conjugated secondary antibodies diluted 1:500 in in FSB-T were added to the sections, and the plates were then incubated for 3 hr at RT in the dark (hereafter, tissue section exposure to light was kept at a minimum to prevent photobleaching). After this incubation period, the secondary antibody solution was removed, and the nuclear counterstain Hoechst-33342 (Thermo Fisher Scientific) was diluted in PBS-T at 1:500 and added to the sections for 10 mins at RT. Subsequently, 4X 5-min washes in PBS-T were carried out. For sequential immunostaining of ZEB1 and MCM2, tissue sections were permeabilised and blocked as above, followed by incubation with rabbit anti-MCM2 diluted 1:500 and chicken anti-GFAP 1:1000 overnight at 4°C. The next day, sections were washed as above and incubated with fluorophore-conjugated secondary antibody diluted 1:500 for 3 hours at RT. After this, sections were washed 3x5 min in PBS-T at RT, followed by blocking FSB-T for 1 hour at RT and another block with anti-rabbit Fab fragments (Jackson ImmunoResearch, 20 μg/ml in FSB-T) for 1 hour at RT. Sections were then incubated with rabbit anti-ZEB1 diluted 1:500 overnight at 4°C. Subsequently, sections were washed 3x5min and incubated with fluorophore-conjugated secondary antibody diluted 1:500 for 1 hour at RT. After this incubation period, the secondary antibody solution was removed, and the nuclear counterstain Hoechst-33342 (Thermo Fisher Scientific) was diluted in PBS-T at 1:500 and added to the sections for 10 mins at RT, followed by 2X 5-min washes in PBS-T. The sections were then mounted onto microscope slides and coverslipped using Prolong Diamond Antifade mountant; these were subsequently allowed to dry at RT, and then stored at 4°C.

For each immunofluorescence sample, one image was taken using a 10X objective for an overview to identify matching sections for the control and Zeb1$^{-/-}$ models. Specifically, the rostral hippocampus at a median bregma −1.8, was used for inter-genotype comparison. For cell quantification, four z stack images (with a z-step of 1-2 μm) were captured spanning the length of the suprapyramidal blade of the dentate gyrus, starting at the inner region of the dentate gyrus and ending at the end of the blade. Images were obtained on a Zeiss LSM710 confocal microscope with Zeiss ZEN software using a 40X (1.3 NA) oil-immersion lens. Subsequently, different cell populations were counted using the Point Tool in ImageJ 1.52K. Where cells were counted within the SGZ, this region was defined as the area covering the height of 2 cell bodies above the boundary between hilus and the granule cell layer. Cell-specific marker expression and morphology were used to determine the inclusion of cells within the counts (Table S2). The primary researcher was not blinded during quantification, but a minimum of one set of technical replicate counts per cell marker per genotype were quantified and confirmed by a secondary blinded researcher.

Clonal analysis

For analysis of individual clones within the DG, mice (n = 6-7 per genotype) were injected with a total dose of 150 μg of Tamoxifen as described above. Clonal recombination was assessed in stereologically sampled sections from animals sacrificed 24 hours after Tamoxifen administration. *Zeb1* deletion was confirmed in clones from Zeb1$^{-/-}$ mice by co-immunostaining for GFAP and ZEB1. Clonal analysis at 24 hours revealed some tdTOM+ neurons with mature morphology at the GCL/ML boundary with no RGL cell in proximity, indicating a low degree of leakiness from the GLAST promoter (Figure S4E). To avoid confounding effects from promoter leakiness, we only quantified clones containing neurons located in the lower half of the GCL. Analysis of clonal progenies was carried out in serial sections from animals sacrificed 4 weeks after Tamoxifen administration. Cells belonging to a clone were identified by proximity, residing within a 90 μm radius of the clone center. Because the GLAST promoter is also active in astrocytes, we excluded astrocytic cells in the hilus, granule cell layer and molecular layer from the analysis. Only astrocytic cells in the SGZ were counted as progenies of RGL cells to exclude confounding effects from recombination in other astrocyte populations. A combination of fluorescent markers and cell morphology was used to identify DG cell types and is presented in Table S2.

Cleavage plane measurements

For quantification of cell division angles, mitotic figures of tdTOM+ cells with RGL morphology were assessed in sections containing the SGZ stained with Hoechst from 7-8 different mice per genotype. ImageJ was used to quantify the cell cleavage angle by drawing a line along the cleavage furrow. A line drawn along the interface between the hilus and SGZ of the DG was used as reference for

cleavage plane angles. Subsequently, the angle measurements were binned into three categories: horizontal (0-30°), intermediate (31-60°), and vertical (61-90°).

Brain slice preparation, electrophysiology and 2-photon imaging

Brain slice preparation and electrophysiology was performed as described previously (Trent et al., 2019). Animals of either sex were deeply anaesthetised using isoflurane, decapitated and their brains removed into chilled (1-3°C) cutting solution containing (in mM) 60 sucrose, 85 NaCl, 2.5 KCl, 1 $CaCl_2$, 2 $MgCl_2$, 1.25 NaH_2PO_4, 25 $NaHCO_3$, 25 D-glucose, 3 kynurenic acid, 0.045 indomethacin. Horizontal hippocampal brain slices (300 m) containing the dentate gyrus, prepared from 8 weeks old Zeb1$^{-/-}$ and control mice 4-5 weeks after tamoxifen injection, were initially stored for 20 minutes at 35°C in sucrose-containing solution and subsequently maintained at room temperature in artificial CSF (aCSF) containing (in mM) 125 NaCl, 2.5 KCl, 1 $CaCl_2$, 1 $MgCl_2$, 1.25 NaH_2PO_4, 25 $NaHCO_3$, 25 D-glucose (305 mOsm) and used within 4-6 hours. For recording, slices were transferred to a submersion chamber continuously perfused with warmed (33-34°C) aCSF containing (in mM) 125 NaCl, 2.5 KCl, 2 $CaCl_2$, 1 $MgCl_2$, 1.25 NaH_2PO_4, 25 $NaHCO_3$, 25 D-glucose (305-10 mOsm, pH 7.4) at a flow rate of 3 ml.min^{-1}. Electrophysiological recordings were performed on dentate gyrus granule cells (DGGC) and of the dentate gyrus granule cell layer. DGGC were identified using Dodt-contrast video microscopy and Tamoxifen-induced cells selected by their expression of tdTOM following 2-photon excitation at l = 900 nm (Prairie Ultima 2-photon microscope, Bruker). Whole-cell current clamp recordings were made using a Multiclamp 700B (Molecular Devices) patch clamp amplifier with patch pipettes with resistances 4–6 MΩ when filled with internal solution containing (in mM) 130 K-gluconate, 20 KCl, 10 HEPES, 0.16 EGTA, 2 Mg-ATP, 2 Na2-ATP, 0.3 Na2-GTP, pH 7.3 (295 mOsm). Somatic series resistance at the start of experiments was between 9-15 MΩ and cells showing changes of R_S greater than 20% over the course of the experiment were rejected. Data were sampled at 20-40 kHz and low-pass filtered at 6 kHz. Resting membrane potential (V_m) was measured as the mean membrane potential during a 100 ms period prior to a hyperpolarizing current injection step averaged across 10-20 sweeps. Input resistance (R_N) was calculated, according to Ohm's law, by dividing the magnitude of the voltage change (sampled over 100 ms) at the end of 1 s hyperpolarizing current injection response by the amount of injected current (20 pA). Membrane time constant (t_m) was measured by fitting a mono-exponential function to the repolarizing phase of the same 20 pA hyperpolarizing current step. Membrane capacitance (C_m) was calculated using t = RC by dividing R_N by t_m.

Neuronal excitability was measured by comparing current injection evoked action potentials in DGGC in Zeb1$^{-/-}$ and control mice. Action potential amplitude, half-width, voltage threshold, dV/dt and rheobase was measured. In order to compare Tamoxifen-induced DGGCs to the larger population of DGGCs patch clamp recordings were performed from both tdTOM+ and tdTOM- cells.

To compare dendritic morphology between Tamoxifen-induced tdTOM+ DGGC in Zeb1$^{-/-}$ and control mice, recorded cells were filled via the recording electrode with Alexa Fluor 488 (100 mM). Stacks of 120-250 2-photon images (512 × 512 pixels) were collected at Z intervals of 1 μm. Soma and dendrites of imaged DGGC were reconstructed post hoc from 3D image projections using the semi-automated tracing tool in Neurolucida 360 (MBF Bioscience). Analysis of dendritic morphology was performed on 3D neuronal reconstructions using Neurolucida Explorer.

In vitro primary cultures and molecular biology

Primary neurosphere cultures were prepared from postnatal day 5 Zeb1$^{-/-}$ mice and cultured in N2 medium with supplemental EGF/FGF2/heparin (20 ng/ml) as previously described (Siebzehnrübl et al., 2018). Briefly, litters of 5-7 mouse pups at postnatal day 5 were sacrificed via cervical dislocation, decapitated and the brain removed and placed in a Petri dish with ice-cold HBSS. The hippocampi were dissected under a stereomicroscope and placed in individual 1.5 mL centrifuge tubes (1 tube per animal) with 1 mL ice-cold HBSS. The tubes were briefly centrifuged at 400 RCF for 3 mins at 4°C to pellet tissue, followed by the aspiration of the supernatant, after which 300 μL PPD solution (0.1% [w/v] Dispase II, 0.01% [w/v] DNase I, 0.01% [v/v] Papain, 12.4 mM $MgSO_4$ in HBSS) was added to each tube for the resuspension and transfer of the tissue solution to a fresh 15 mL tube. The tissue was incubated in PPD solution at 37°C for 15 mins, with gentle trituration (6 times) with a sterilized fire-polished glass pipette every 5 mins during the incubation period. A 70 μM cell strainer (one per cell suspension tube) was prepared by pre-wetting with 1 mL ice-cold 1XPBS, followed by straining of the cell suspension, and a further wash with 10 mL ice-cold 1X PBS to remove residual cells in the strainer. The cell suspension was transferred to a fresh 15mL tube and centrifuged at 400 RCF for 5 mins at 4°C, followed by the aspiration of the supernatant. The pelleted cells were resuspended in 10 mL ice-cold 1XPBS and centrifuged again as before. After the aspiration of the supernatant, the cell pellet was resuspended in N2 medium supplemented with growth factors (EGF, FGF2, heparin; final concentrations 20 ng/ml) and plated in 12-well plates, pre-coated with 100 μg/ml poly-D-lysine and 5 μg/ml laminin. As the genotype of pups could not be determined prior to preparing cell cultures, cells from individual pups were plated into separate wells and once the genotype was determined, cell cultures with the same transgene status were pooled at the time of first passage. 50% of medium was replaced the day after cell isolation, and growth factors were supplemented in the medium again; thereafter, the cells were passaged as detailed below, with growth factors supplemented every two days.

All cells were maintained at 37°C in a humidified incubator with 5% CO_2. Neurospheres were passaged approximately every 7-10 days. For this, spheres were collected in a 15 mL tube and centrifuged at 400 RCF for 5 mins. The supernatant was aspirated and replaced with 500 μL Accumax and incubated at 37°C for 10 mins. Subsequently, 9.5 mL 1XPBS was added to the tube and the cell suspension was centrifuged at 400 RCF for 5 mins. The supernatant was removed, and the cells were resuspended in

200 μL N2 medium and triturated to achieve a single cell suspension. $1x10^5$ cells/mL of N2 medium were seeded as above. Neurospheres between passage 2 and 4 were used for downstream experiments.

To induce recombination, primary cultures were treated with 4-hydroxy-Tamoxifen (10 μM) for 24 hours after which the culture medium was removed and replaced with fresh N2 supplemented with growth factors. For ETV5 rescue experiments, some cultures were transduced with lentiviral particles carrying an *Etv5* expression cassette (pLX_TRC311 ETV5, gift from William Hahn; Wang et al., 2017).

ChIP analysis was performed using the SimpleChIP kit (Cell Signaling) according to the manufacturer's instructions. $4x10^6$ cells were plated in a 75 cm^2 flask for each immunoprecipitation, testing for a negative control (IgG) and $Zeb1^{-/-}$ cells. Per immunoprecipitation sample, 10 μg of cross-linked chromatin was used, with the addition of 2 μg appropriate antibody per sample.

Protein extraction and Western Blot were performed as described (Jimenez-Pascual et al., 2019). Antibodies used for ChIP and Western Blot are provided in the Key resources table.

In vitro time-lapse imaging of primary hippocampal cells

Primary SGZ cells were prepared from the rostral half of the hippocampus of adult GLAST::$CreER^{T2}$, $Rosa26^{lox\text{-}stop\text{-}lox\text{-}tdTomato}$ (n = 4) and GLAST::$CreER^{T2}$, $Rosa26^{lox\text{-}stop\text{-}lox\text{-}tdTomato}$, $Zeb1^{flox/flox}$ (n = 7) mice. Tissue was dissected in ice-cold HBSS with magnesium (with 1% HEPES). After the hippocampus was dissected, tissue was incubated at 37°C for 25 minutes in a dissociation solution (HBSS-based, with 0.5% glucose, 1.25% HEPES) with Hyaluronidase from bovine testes (7 mg/10 ml) and Trypsin from bovine pancreas (> 7.500 BAEE units/mg, 7 mg/10 ml). Tissue was triturated once during the incubation. Digestion was stopped by adding equal volume of ice-cold EBSS-based solution (with 4% Bovine Serum Albumin and 2% HEPES). Cell suspension was filtered through 40 μm cell strainer and centrifuged. Cell pellet was resuspended in ice-cold 1 mL DMEM/F12 with Glutamax and pipetted on the top of 10 mL of the ice-cold EBSS-based solution for gradient centrifugation. Cell pellet was dissociated in 1 mL of a cell maintenance medium containing DMEM/F12 with Glutamax with 1% Penicillin-Streptomycin (10.000 U/ml), 2% B27 cell supplement and 5 ng/ml of EGF and FGF2. 500 μl of this cell suspension was plated on PDL-coated wells in a standard 24-well cell culture plate. Cells were kept at 37°C and 5% CO_2 in a humified cell incubator. After 6 hours, cells were washed with DMEM/F12 and exposed to 4-hydroxy-Tamoxifen (10 μM) in the cell maintenance medium for 16 hours to induce genetic recombination. In some experiments (n = 7), cell cultures from GLAST::$CreER^{T2}$, $Rosa26^{lox\text{-}stop\text{-}lox\text{-}tdTomato}$, $Zeb1^{flox/flox}$ animals were transduced with lentiviral particles carrying *Etv5* expression cassette concurrently with 4-hydroxy-Tamoxifen. After incubation, cells were washed with DMEM/F12 and kept in the maintenance medium. 24 hours after incubation, cells were placed in an Pecon environmental chamber of a time-lapse imaging set-up based on the Zeiss Axio Observer 7 inverted microscope with the Zeiss Axiocam 705 camera and a motorized, programmable stage. During the time-lapse imaging, cells were kept at 100% humidity, 37°C and 5% CO_2. The time-lapse imaging of adult neural stem cells was based on previously described protocol (Ortega et al., 2013; Petrik et al., 2018). In each well, 4-7 tile-clusters (2x2, 3482 × 3906 pixels) were continuously imaged in the brightfield every 10 minutes for 6 days using an apochromat 10X objective (NA = 0.45). Imaging used automated z axis focus correction by Zeiss ZEN definitive focus function. Imaging operation and acquisition was managed by the Zeiss ZEN Blue software. Each imaging tile cluster was saved as an individual file. After imaging, cells were immunostained for GFAP (rabbit, 1:400), DCX (guinea pig, 1:400) and Ki67 (1:300) overnight at room temperature and for 2 hours with fluorophore-conjugated secondary antibodies. Fluorescence images of the tile clusters from the time-lapse images were taken with the 10X objective for a post hoc identification of cell types. Cell dynamics was analyzed directly in the ZEN software in cases of isolated and rarely dividing cell clones or converted by the tTt converter and traced in The Tracing Tool (tTt) software (Hilsenbeck et al., 2016) in more densely populated or intensely dividing cell clones. Number of cells and cell divisions per clone and the length of cell cycle for each cell division were recorded. To distinguish symmetric versus asymmetric cell divisions, we defined an asymmetric cell division as a division where one daughter cell continued to divide, and the other daughter cell did not divide for at least 1.5 times the time from the last cell division or longer. Non-dividing cell clones were defined as individual adult neural stem cells with radial glia-like morphology that did not divide for the entire length of the time-lapse experiment.

QUANTIFICATION AND STATISTICAL ANALYSIS

Statistical testing was carried out using GraphPad Prism 9.1. Normal distribution of values was tested using a D'Agostino & Pearson test. For comparison of two groups, two-tailed t tests were used for normally distributed data and Mann-Whitney tests where data was not normally distributed. For comparison of 3 or more groups, one-way ANOVA (with Fishers LSD test) was used for normally distributed data and Kruskal-Wallis tests (with Dunn's test) where data was not normally distributed. Grouped data were analyzed using two-way ANOVA and Fishers LSD test. For categorical analyses, a Chi square test was used. P values of individual statistical analyses are presented in the figures. A p value of less than 0.05 was deemed significant. Unless otherwise specified, data are presented as mean ± SEM.

Cell Reports, Volume *36*

Supplemental information

The transcription factor ZEB1 regulates stem cell self-renewal and cell fate in the adult hippocampus

Bhavana Gupta, Adam C. Errington, Ana Jimenez-Pascual, Vasileios Eftychidis, Simone Brabletz, Marc P. Stemmler, Thomas Brabletz, David Petrik, and Florian A. Siebzehnrubl

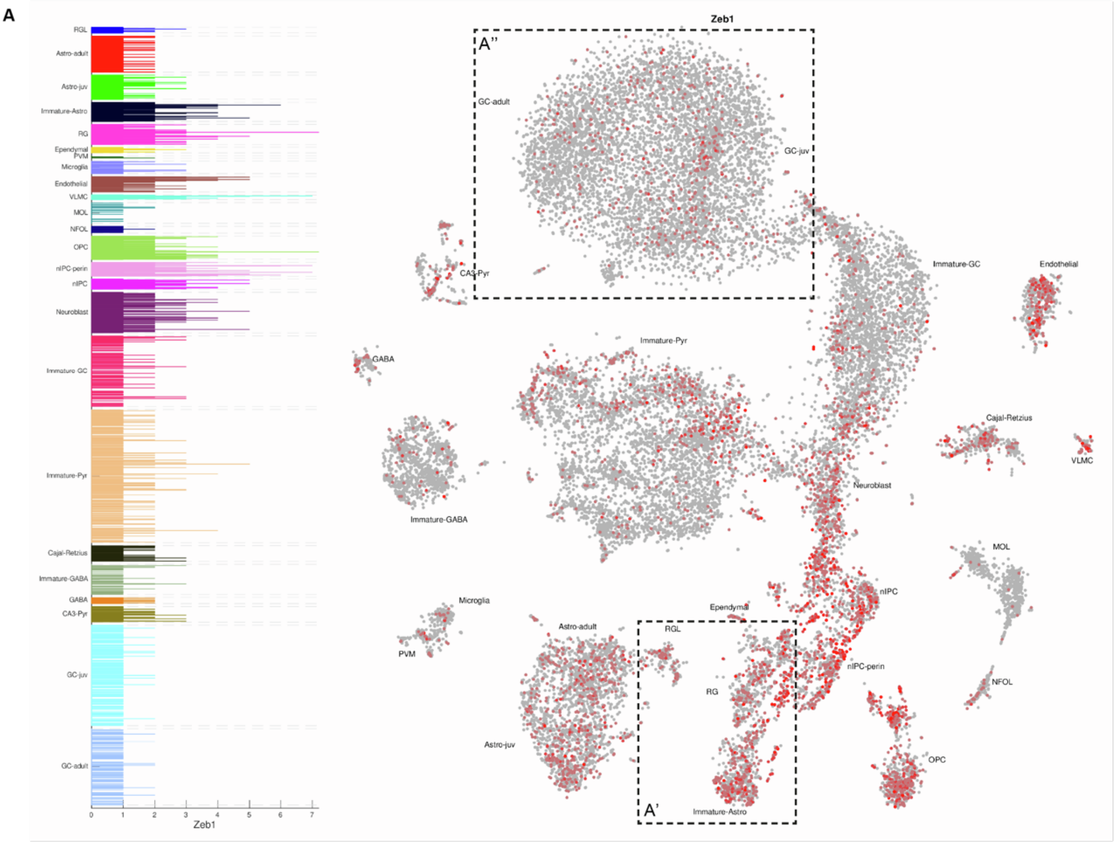

Figure S1: Single-cell RNA-Sequencing data of *Zeb1* expression across hippocampal cell types. Related to Figure 1. Depicted is data from Hochgerner et al. (Hochgerner et al., 2018) based on 10X Genomics sequencing. Boxed areas indicate RGL cell and immature astrocyte populations **(A')** as well as hippocampal granule cells **(A'')**.

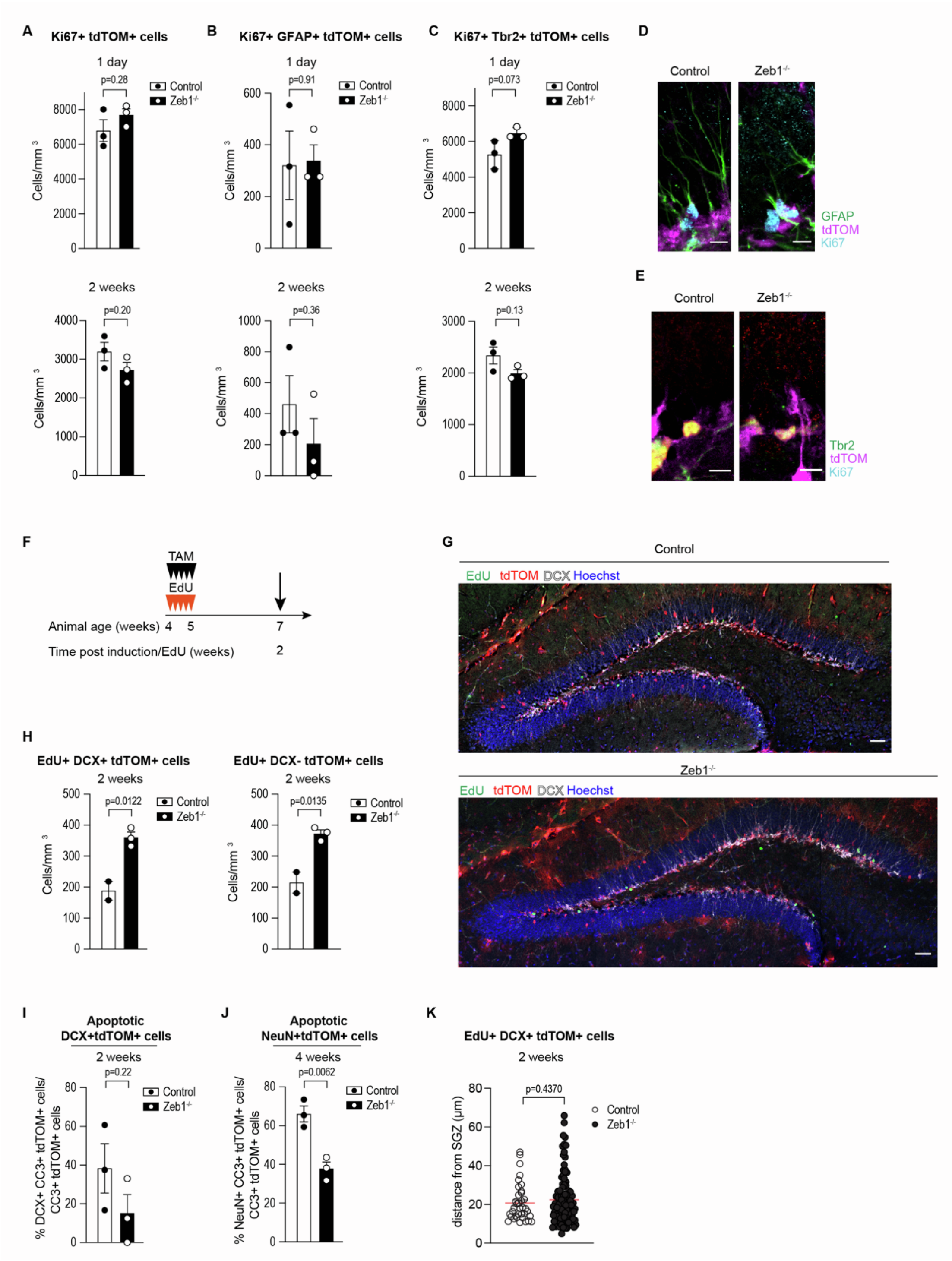

Figure S2: *Zeb1* deletion does not result in slower proliferation rates but specifically increases neuronal survival. Related to Figure 4. (A) Quantification of proliferating Ki67+ cells in control and *Zeb1*$^{-/-}$ mice. Both genotypes show no significant differences at 1 day or 2 weeks post induction. **(B)** Quantification of proliferating Ki67+GFAP+tdTOM+ RGL cells in control and *Zeb1*$^{-/-}$ mice. Both genotypes show no significant differences at 1 day or 2 weeks post induction. **(C)** Quantification of proliferating Ki67+Tbr2+tdTOM+ IPCs in control and *Zeb1*$^{-/-}$ mice. Both genotypes

show no significant differences at 1 day or 2 weeks post induction. **(D-E)** Representative images of Ki67+GFAP+tdTOM+ cells **(D)** and Ki67+Tbr2+tdTOM+ at 2 weeks post-induction **(E)**. Scale bars 10 µm. **(F)** Schematic of EdU administration concurrent with tamoxifen; tissue harvesting was carried out at 2 weeks post-EdU injections. **(G)** Representative images of hippocampi stained for EdU, DCX and tdTOM. Scale bars 100 µm. **(H)** Quantification of EdU+ cells showed a significant increase of both EdU+tdTOM+DCX+ (left) and EdU+tdTOM+DCX- (right) cells 2 weeks after EdU injection. **(I)** Quantification of the fraction of apoptotic CC3+ neuroblasts (DCX+) showed no difference between the genotypes. **(J)** Quantification of the fraction of apoptotic CC3+ neurons (NeuN+) demonstrated a decrease in the number of apoptotic neurons in the *Zeb1*$^{-/-}$ mice in comparison to controls at 4 weeks post-induction. **(K)** Distances of EdU+tdTOM+DCX+ neuroblasts from the SGZ show no significant differences in migration of newborn neuroblasts between genotypes. Dots represent individual animals, except in K where dots represent individual cells.

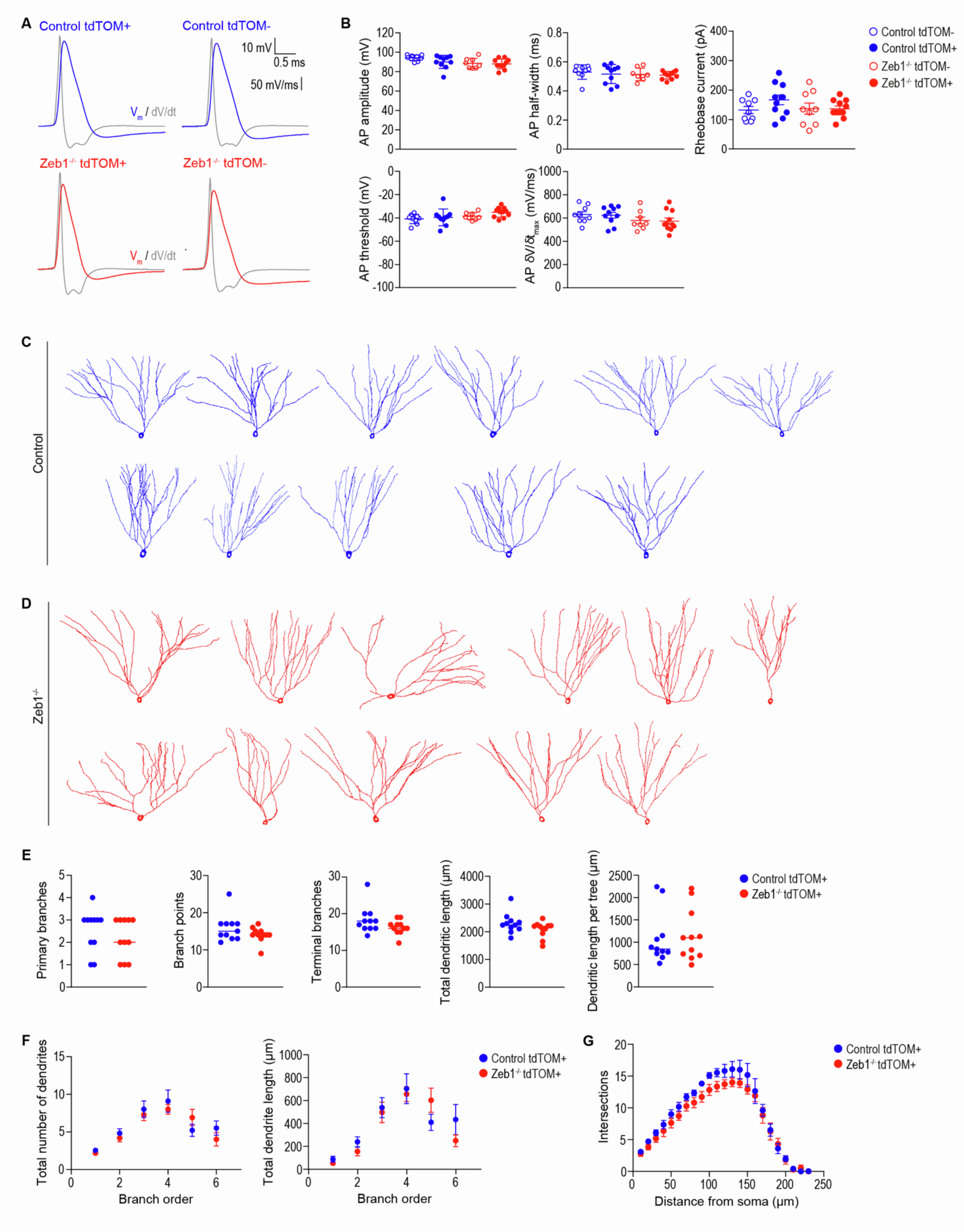

Figure S3: Patch-clamp electrophysiology of control and *Zeb1*$^{-/-}$ neurons. Related to Figure 4.

(A) Traces showing evoked action potentials, in tdTOM+ and tdTOM-, control (blue) and *Zeb1*$^{-/-}$ (red) DGGCs. Grey traces show the first temporal derivative (δV/δt) of the action potential. **(B)** Scatter

plots showing action potential amplitude, half width, voltage threshold, δV/δt and rheobase current for individual DGGCs overlaid with the mean and SEM value for each group. (**C-D**) 2D projections of reconstructed DGGCs from control **(C)** and Zeb1$^{-/-}$ mice (**D**, n = 11 each). **(E-F)** Plots of the mean number of dendrites and mean total dendritic length by branch order in control and *Zeb1*$^{-/-}$ DGGCs. **(G)** Sholl plot of the number of dendritic intersections against distance from the soma. Dots represent individual neurons in B and E, and averages in F and G.

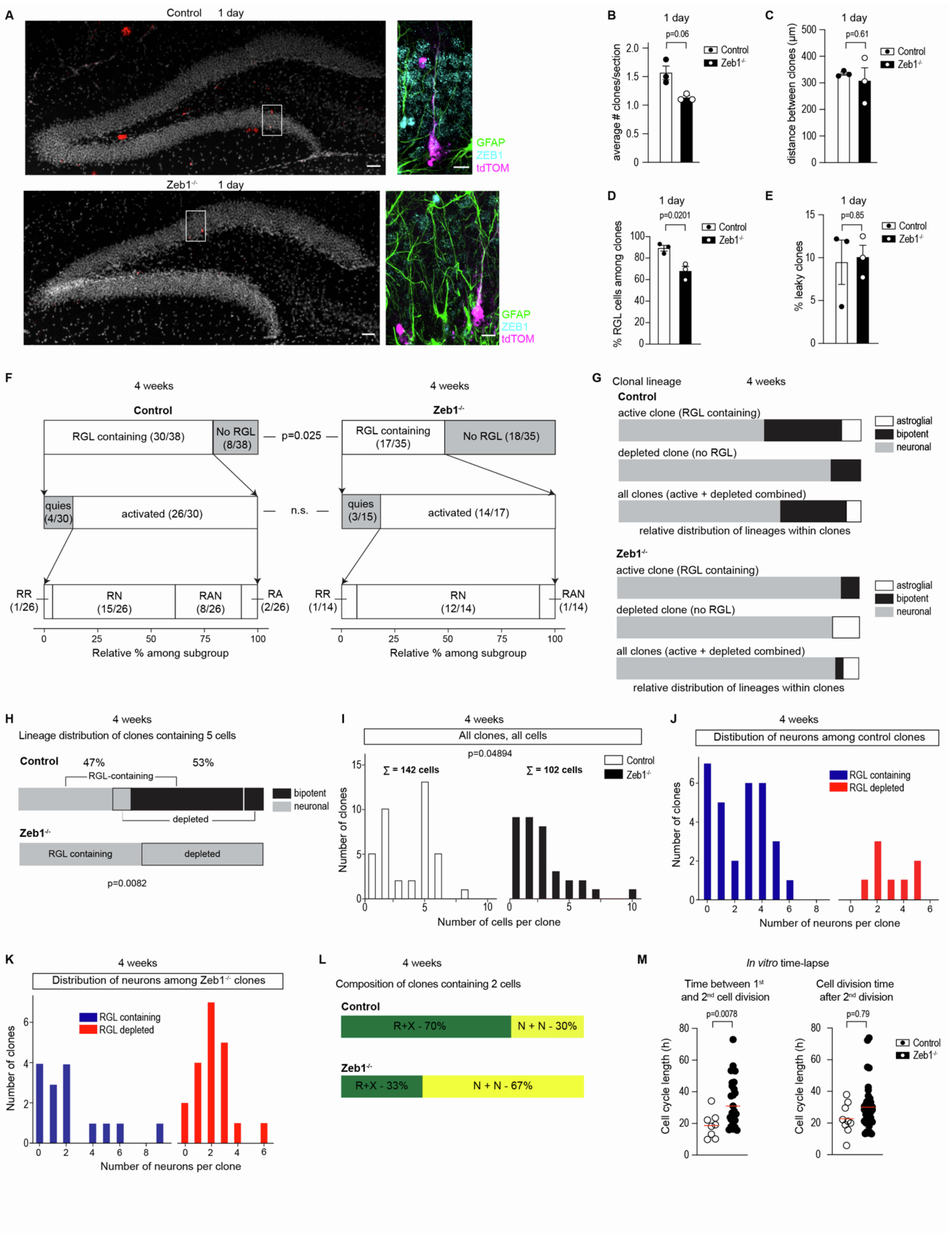

Figure S4: Additional data from clonal analysis. Related to Figure 6. (A) Representative images of hippocampi 1 day after administration of low-dose tamoxifen. Insets show recombination of tdTOM in RGL cells (ZEB1+ in control, ZEB1- in *Zeb1*$^{-/-}$). Note the presence of the tdTOM+ IPC in proximity

to the RGL cell in *Zeb1*$^{-/-}$. Recombined clones in *Zeb1*$^{-/-}$ mice displayed evidence of accelerated proliferation compared to controls (see also D). Scale bars 100 µm (10 µm in insets). **(B)** Quantification of recombined clones per section 1 day after induction shows no difference between genotypes. **(C)** Average distance between recombined clones (where there was more than 1 clone per section) shows no difference between genotypes. **(D)** Quantification of numbers of RGL cells per clone shows a significant decrease in *Zeb1*$^{-/-}$ mice compared to controls at 1 day post induction. **(E)** Quantification of leaky clones (containing >4 cells at 1 day post induction) shows no difference between genotypes. **(F)** Comparison of clonal distribution shows significantly fewer active (RGL cell-containing) clones in *Zeb1*$^{-/-}$ mice. Among RGL-containing (active) clones, there is no difference in the ratio of quiescent (containing only a single RGL cell) to activated (containing an RGL cell and any other cell) clones. Lineage distribution of progenies in activated clones showed a profound loss of astroglial cells in *Zeb1*$^{-/-}$ clones (R: RGL cell; RR: self-renewing clone containing 2 RGL cells; RN: clone containing RGL cell and neuronal progenies; RA: clone containing RGL cell and astroglial progenies; RAN: clone containing RGL cell, neuronal and astroglial progenies). **(G)** Comparison of lineage distribution across active, depleted and all clones highlights increase in neuron-producing and decease in astrocyte-producing clones in *Zeb1*$^{-/-}$ mice. No difference was found between the percentages of neuronal lineage producing depleted clones, but these were far fewer in total numbers in control mice. **(H)** Comparison of lineage distribution across clones containing 5 cells. In controls, 53% of 5-cell clones are bipotent (producing neuronal and astroglial progenies), whereas in Zeb1$^{-/-}$ mice all 5-cell clones were committed to the neuronal lineage. In controls, the majority of 5-cell clones contained an RGL cell, but in *Zeb1*$^{-/-}$ mice the number of active and depleted 5-cell clones was approximately even. **(I)** Total number of cells per clone was significantly lower in *Zeb1*$^{-/-}$ clones compared to controls. Note the different distribution of cells among clones between controls (peaks at clones containing either 2 or 5 cells) and *Zeb1*$^{-/-}$ mice (left shift towards clones containing 1-3 cells). **(J,K)** Histograms showing distribution of neurons between RGL-containing and RGL-depleted clones for control **(J)** and Zeb1$^{-/-}$ mice **(K)**. In controls, most clones contain an RGL cell with peaks at 1 and 4 neurons (corresponding to peaks at 2 and 5 cells in panel D), whereas most *Zeb1*$^{-/-}$ clones lack an RGL cell with a single peak around 2 neurons. **(L)** Comparison of lineage distribution across clones containing 2 cells. In control mice, most 2-cell clones are activated,

whereas in *Zeb1*$^{-/-}$ mice the majority of 2-cell clones is depleted and contains neurons. **(M)** Quantification of cell division times between first and second division (left) and all subsequent divisions (right) from time-lapse imaging. Red line represents median. Dots represent individual animals in B-E and individual clones in M.

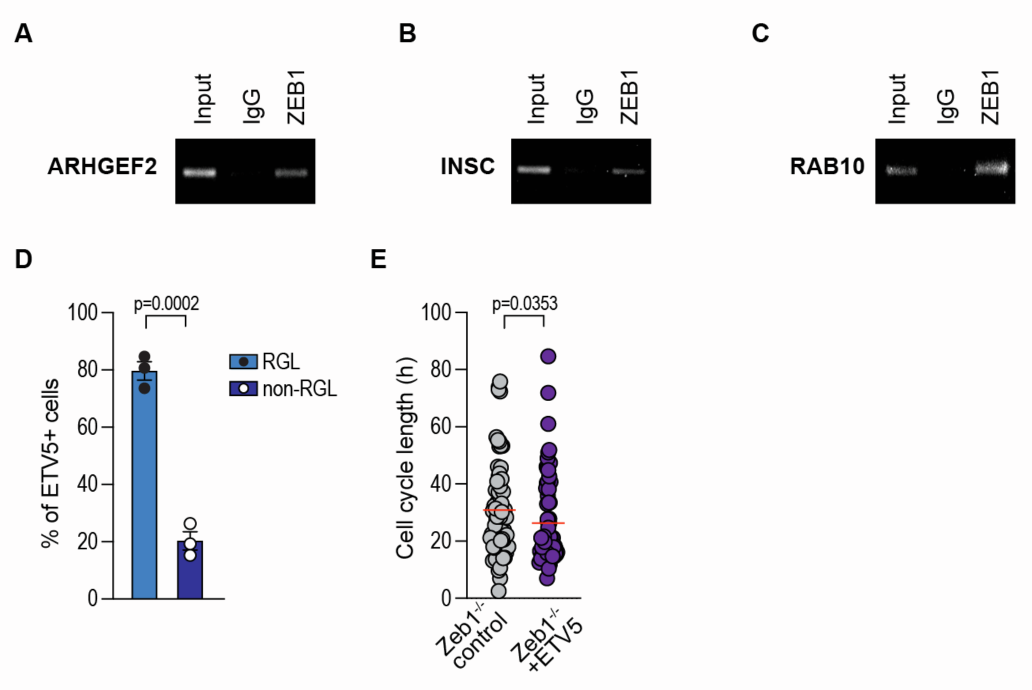

Figure S5: ChIP data for promoter occupancy of ZEB1. Related to Figure 7. (A-C) ChIP-PCR demonstrated ZEB1 binding to the promoter regions of ARHGEF2 **(A)**, INSC **(B)** and RAB10 **(C)**. **(D)** Quantification of ETV5 expression across cell types in the SGZ shows that the majority of ETV5 expressing cells are RGL cells, with a small fraction of non-RGL ETV5+ cells. **(E)** Quantification of cell cycle length in *Zeb1*$^{-/-}$ control and ETV5-transduced clones. Red line represents median. Dots represent individual animals in D and individual cells in E.

Table S1: Candidate regulators of asymmetric cell division. Candidates included in further analyses are highlighted. Related to Figure 7.

**Candidate gene name**	ACTR2	ACTR3	ARHGEF2	ASPM	DOCK7	ETV5	FGF13	GOLGA2	HOXC4	ING2	INSC	PARD3	PAX6	POU5F1	RAB10	RGS14	SOX5	STRA8	TEAD3	WNT9B	ZBTB16
ZEB1 bound in GBM ChIP-Seq?		♦	♦			♦			♦	♦	♦	♦			♦		♦				
Expression in HC cell types (1)	neuron	neuron	IPC/ neuron	IPC	astro, neuron	RGL, astro, OPC	neuron	broad		broad	neuroblast	broad	RGL, astro, neuroblast		broad	granule cell	broad				Neuron
Expression in astrocytes (2)				♦ (STR)		♦ (HC)					♦ (STR)	♦ (STR)	♦ (HC)								♦ (HC)
Cell types showing overlap with ZEB1 (3)	oligo	oligo	astro/OPC		oligo	astro/OPC	oligo	astro/ OPC		oligo	astro	astro	astro		astro/ oligo	oligo	astro/ OPC				astro/ OPC

(1) Hochgerner et al. Nat Neurosci 2018 DOI: 10.1038/s41593-017-00562-2
(2) Chai et al. Neuron 2017 DOI: 10.1016/j.neuron.2017.06.029
(3) Zeisel et al. Science 2015 DOI: 10.1126/science.aaa1934

Acronyms:
astro = astrocyte
GBM = glioblastoma
HC = hippocampus
IPC = intermediate progenitor cell
oligo = oligodendrocyte
OPC = oligodendrocyte precursor cell
RGL = radial glia like cell
STR = striatum

Table S2: Criteria for identifying cell types in the DG. Related to STAR Methods sections 'Tissue processing, immunostaining, and confocal imaging' and 'Clonal analysis'.

Cell population	Markers	Localization/Morphological features
RGL cell	GFAP	SGZ-localized soma, a radial projection spanning the GCL and branching in ML
Activated RGL, early IPC	MCM2	SGZ-localized, either GFAP+ RGL cell-like morphology or spherical morphology and GFAP- (IPC)
Proliferating IPC	Ki67	SGZ-localized, spherical
Late IPC	TBR2	SGZ-localized, spherical with some short processes
Neuroblast, immature neuron	DCX	SGZ and lower GL-localized, short process extending towards upper GCL
Mature neuron	NeuN	Pan-DG localization with a projection branching in the ML
Astrocyte	GFAP, S100ß	SGZ, hilus, and ML-localized with a stellate morphology

Table S3: Oligonucleotide information, related to STAR Methods.

Reagent or Resource	Source	Identifier
ARHGEF fwd #1 (ChIP)	Sigma-Aldrich	TCTGGCTTGTGTGGCTGAAA
ARHGEF rev #1 (ChIP)	Sigma-Aldrich	TCACAAATCAGAGCCCGGTT
ARHGEF fwd #2 (ChIP)	Sigma-Aldrich	GTCTGTGTGGGCAAAACACG
ARHGEF rev #2 (ChIP)	Sigma-Aldrich	AGCTAATCCCAACTGGAGCC
ETV5 fwd #1 (ChIP)	Sigma-Aldrich	ATTCAGGCAAGAGGGGGAGT
ETV5 rev #1 (ChIP)	Sigma-Aldrich	ACAACATACTGGGCGAGCCA
ETV5 fwd #2 (ChIP)	Sigma-Aldrich	TGCACAATGGGGTCTGGTTA
ETV5 rev #2 (ChIP)	Sigma-Aldrich	CCCCTCTTGCCTGAATGGTG
INSC fwd #1 (ChIP)	Sigma-Aldrich	TCCAGAGAGTACAGGCCACA
INSC rev #1 (ChIP)	Sigma-Aldrich	TCACCAATACCACGCCAGAG
INSC fwd #2 (ChIP)	Sigma-Aldrich	GAAGCACATGCTGATCTGTGG
INSC rev #2 (ChIP)	Sigma-Aldrich	AGGAAGCCGCCTCACCAATA
PARD3 fwd #1 (ChIP)	Sigma-Aldrich	GAGCTCCACCTGCCTTTGAC
PARD3 rev #1 (ChIP)	Sigma-Aldrich	AAAGAAGTTGTGCCTGGCTTG
RAB10 fwd #1 (ChIP)	Sigma-Aldrich	GGCCTCCTGATTGGGTGAG
RAB10 rev #1 (ChIP)	Sigma-Aldrich	CTATACGGACGTAGTGCGCC
RAB10 fwd #2 (ChIP)	Sigma-Aldrich	AGAGCCACCTTTTTCCCCGT
RAB10 rev #2 (ChIP)	Sigma-Aldrich	GGTGTACGTCCTTGCGTGC
SOX5 fwd #1 (ChIP)	Sigma-Aldrich	AGCGAAGTGGAGCTATGACA
SOX5 rev #1 (ChIP)	Sigma-Aldrich	GAGCGCTCTCAGCAGTAAGT
SOX5 fwd #2 (ChIP)	Sigma-Aldrich	TGGAGCTATGACATCCCACAT
SOX5 rev #2 (ChIP)	Sigma-Aldrich	AGCGCTCTCAGCAGTAAGTA
